# The biphasic role of Hspb1 on ferroptotic cell death in Parkinson's disease

**DOI:** 10.7150/thno.98457

**Published:** 2024-08-01

**Authors:** Jieyi Meng, Jinyu Fang, Yutong Bao, Huizhu Chen, Xiaodan Hu, Ziyuan Wang, Man Li, Quancheng Cheng, Yaqiong Dong, Xiaoda Yang, Yushu Zou, Dongyu Zhao, Jiping Tang, Weiguang Zhang, Chunhua Chen

**Affiliations:** 1 Department of Anatomy and Embryology, School of Basic Medical Sciences, Peking University Health Science Center, Beijing 100191, China.; 2 School of Clinical Medicine, Peking University Health Science Center, Beijing 100191, China.; 3 Institute of Translational Medicine, College of Medicine, Qingdao University, Qingdao, Shandong 266023, China.; 4 The State Key Laboratories of Natural and Biomimetic Drugs and Department of Chemical Biology, School of Pharmaceutical Sciences, Peking University Health Science Center, Beijing 100191, China.; 5 Department of Biomedical Informatics, School of Basic Medical Sciences, Peking University, Beijing, 100191, China.; 6 State Key Laboratory of Vascular Homeostasis and Remodeling, Peking University, Beijing, 100191, China.; 7 Physiology and Pharmacology Department of Basic Sciences, Loma Linda University School of Medicine, Loma Linda 92350, USA.

**Keywords:** Parkinson's disease, ferroptosis, Nrf2, transcriptional regulation, Hspb1

## Abstract

**Rationale**: Ferroptosis-driven loss of dopaminergic neurons plays a pivotal role in the pathogenesis of Parkinson's disease (PD). In PD patients, Hspb1 is commonly observed at abnormally high levels in the substantia nigra. The precise consequences of Hspb1 overexpression in PD, however, have yet to be fully elucidated.

**Methods**: We used human iPSC-derived dopaminergic neurons and Coniferaldehyde (CFA)—an Nrf2 agonist known for its ability to cross the blood-brain barrier—to investigate the role of Hspb1 in PD. We examined the correlation between Hspb1 overexpression and Nrf2 activation and explored the transcriptional regulation of Hspb1 by Nrf2. Gene deletion techniques were employed to determine the necessity of Nrf2 and Hspb1 for CFA's neuroprotective effects.

**Results**: Our research demonstrated that Nrf2 can upregulate the transcription of Hspb1 by directly binding to its promoter. Deletion of either Nrf2 or Hspb1 gene abolished the neuroprotective effects of CFA. The Nrf2-Hspb1 pathway, newly identified as a defense mechanism against ferroptosis, was shown to be essential for preventing neurodegeneration progression. Additionally, we discovered that prolonged overexpression of Hspb1 leads to neuronal death and that Hspb1 released from ruptured cells can trigger secondary cell death in neighboring cells, exacerbating neuroinflammatory responses.

**Conclusions**: These findings highlight a biphasic role of Hspb1 in PD, where it initially provides neuroprotection through the Nrf2-Hspb1 pathway but ultimately contributes to neurodegeneration and inflammation when overexpressed. Understanding this dual role is crucial for developing therapeutic strategies targeting Hspb1 and Nrf2 in PD.

## Introduction

Parkinson's disease (PD) is characterized by the progressive loss of dopaminergic neurons in the substantia nigra 1. This neuronal loss is primarily attributed to mitochondrial dysfunction and the buildup of Lewy bodies, which consist of misfolded α-synuclein aggregates 2. These pathological alterations trigger a series of cell death pathways, encompassing parthanatos, necroptosis, pyroptosis, and apoptosis, contributing to the disease's progression 3-6. Besides these, ferroptosis is another significant mode of programmed cell death, characterized by its unique mechanisms. Ferroptosis is distinguished by the accumulation of lipid peroxidation, driven by cysteine depletion and elevated intracellular labile iron levels. The process begins as reactive oxygen species (ROS) attack polyunsaturated fatty acids in lipid bilayers, leading to the formation of phospholipid hydroperoxides and subsequent plasma membrane disruption, resulting in cell death. This chain reaction disrupts the integrity of the plasma membrane, ultimately leading to cell death 7-10. Key regulators of iron homeostasis and lipid peroxidation such as gp91^phox^, LOX, FPI-1, FPI-2, Gpx4, Slc7a11, and Acsl4 play critical roles in the initiation and progression of ferroptosis 11-14. This mode of cell death has been pinpointed as a potential therapeutic target in neurodegenerative conditions including Alzheimer's disease, Parkinson's disease, and multiple sclerosis 15-17. Modulating these ferroptosis-related genes could offer a strategy to protect dopaminergic neurons, potentially slowing down the progression of PD.

Several studies have identified Nrf2 (nuclear-factor-E2-related factor 2, also known as NFE2L2), a key antioxidant transcription factor, as a promising therapeutic target for PD 18-20. Nrf2 plays a crucial role in maintaining iron and glutathione (GSH) homeostasis and mitigating lipid peroxidation by regulating the expression of anti-ferroptosis genes such as Sod2, Hmox1, Mrp1, Gpx4, Slc7a11 and H-ferritin 21-26. In our preliminary study, we evaluated the cytoprotective and anti-ferroptosis properties of three Nrf2 activators—Coniferaldehyde (CFA), Sulforaphane (SFN), and 4-Octyl Itaconate (4OI) in vitro 27-34. We specifically focused on CFA, due to its efficient blood-brain barrier permeability and low cytotoxicity 27. Previous studies have demonstrated that CFA was significant effectiveness in combating Alzheimer's disease by activating Nrf2 and enhancing the clearance of amyloid-beta (Aβ) in the brain 29. In this study, RNA sequencing data indicated a correlation between elevated expression of Hspb1 and activation of Nrf2 in PD.

Hspb1, a member of the small heat-shock proteins (sHSPs) family, serves primarily as a molecular chaperone. It plays a pivotal role in stabilizing protein scaffolds, such as the SQSTM1 complex 35, and in mitigating α-synuclein aggregation 36. Furthermore, Hspb1 is pivotal in modulating protein quality control to maintain mitochondrial homeostasis 37, 38. In addition, it has the ability to translocate into the nucleus, where it interacts with proteins in nucleus to enhance cellular resistance to oxidative stress and inhibit cell death 39-41. In various neurodegenerative diseases, including Alzheimer's disease (AD), PD, multiple sclerosis (MS), amyotrophic lateral sclerosis (ALS) and frontotemporal dementia, Hspb1 has been consistently observed to be upregulated, highlighting its significance as a key molecule of interest 42-46. Studies have also indicated that overexpression of Hspb1 could inhibit both pyroptosis and apoptosis 47, 48, whereas its depletion leads to reduced resistance against ER stress and proteotoxic stress 49, 50. These findings suggest that the upregulation of Hspb1 may inhibit cell death, making it a potential therapeutic target for the treatment of neurodegenerative diseases. Recent advancements in research on ferroptosis have revealed that Hspb1 overexpression inhibits erastin-induced ferroptosis in tumors 51. However, the link between increased Hspb1 expression and ferroptosis in PD remains underexplored. In our study, we observed a transient increase in Hspb1 in response to neuronal oxidative stress, which effectively decreased lipid peroxidation accumulation and inhibited ferroptosis in neurons. We also discovered a significant correlation between Hspb1 expression and Nrf2 activation, a linkage previously reported in Caenorhabditis elegans but not yet confirmed in mammalian models 52-54.

Our further investigations into Hspb1 have revealed a paradox. Although initial elevation of this protein provides neuronal protection, prolonged overexpression eventually results in significant neuronal death. This paradox is also evident in myocardial injury studies, indicating a dual role for Hspb1 depending on its cellular location. Intracellularly, Hspb1 serves a protective function. In contrast, when Hspb1 is present extracellularly, it activates the TLR2/4 pathway, triggering an NFκB-mediated inflammatory response that contributes to cellular damage 55. This extracellular presence of Hspb1 often occurs in certain disease states, and it has been identified as a potential biomarker for clinical diagnostics 56-58 Under normal conditions, Hspb1 is minimally expressed in neurons and microglia and is not released via the canonical ER-Golgi secretion pathway. Instead, it is released during cell death processes such as necrosis, which involve membrane rupture, leading to the extracellular accumulation of pathologically overexpressed Hspb1 59-61.

Given the complex role of Hspb1, we conducted both in vivo and in vitro experiments to elucidate its function in the pathogenesis and progression of PD. By understanding how Hspb1 influences neuronal survival and its involvement in inflammatory pathways, we aim to better target interventions that modulate its expression or mitigate its harmful effects.

## Materials and methods

### Mice

In this study, we adhered to ethical standards, minimizing the number of mice used and alleviating their distress. Mice were closely monitored with daily body weight assessments. All animal experiments were approved by the Peking University Animal Ethics Committee and followed the guidelines of the Institutional Animal Care and Use Committee (IACUC) of Peking University. Nrf2^-/-^ mice with a C57BL/6 background were kind gifts from Dr. Siwang Yu of Peking University.

### Agent treatments

After a one-week acclimatization, male mice (8 weeks old, 24 ± 0.3 g) received 4 intraperitoneal injections of MPTP-HCl (23.4mg/kg) at 2-h intervals over a single day 62. Control groups were administered an equivalent volume of 0.9% saline solution. Subsequently, the mice were treated with various doses of Coniferaldehyde (CFA) dissolved in DMSO (0.1, 1, 10, 20, and 50 mg/kg) for a duration of 14 days. To elucidate the neuroprotective mechanism of CFA in PD, we established both in vivo and in vitro PD models using the neurotoxic compound 1-methyl-4-phenyl-1, 2, 3, 6-tetrahydropyridine (MPTP) and its active metabolite 1-methyl-4-phenylpyridinium (MPP^+^). MPTP, a highly lipophilic compound, can cross the blood-brain barrier, and metabolize to MPP^+^ in glial cells. MPP^+^ is then released into the extracellular matrix, and subsequently taken up by DAergic neurons, where it accumulates in the mitochondria. MPP^+^ inhibits Complex I of the mitochondrial respiratory chain, generating toxic ROS that leads to cell death 63, 64. MPTP exposure induces PD-like symptoms including motor deficits, dopamine depletion, and α-syn accumulation 65, 66. The reagents utilized in this study are detailed in Table S1.

### Behavior tests

All behavior tests were performed in a quiet environment with a constant room temperature maintained at around 25°C. The tests in the MPTP mouse model of PD were evaluated on Day 15-16 after CFA administration.

The pole test was conducted using a 75 cm long rod (diameter = 0.9 cm) wrapped with a gauze bandage. Mice were placed near the top of the pole facing upwards, and the time taken to touch the bottom was recorded. Prior to MPTP injection, mice were trained for 2 consecutive days. The actual test was performed on day 15, consisting of 3 trial runs. The shortest time recorded for each mouse was used for analysis. For the rotarod test, a rotarod machine equipped with automatic timers and fall sensors was used. Mice were placed on an accelerating rod (accelerated from 4 to 40 rpm over 5 minutes), and the latency to fall was recorded. Each mouse underwent 3 trials, and the longest time was used for analysis. Mice were trained for two consecutive days before the MPTP injection. The wire hanging test evaluates muscle strength and motor coordination. Mice were gently placed on an iron wire (20 mm in diameter, 70 cm above the floor). A curved plastic safety liner was placed under the wire to prevent injuries. The latency to fall was recorded, and the longest time was used for further analysis. Each mouse was tested 3 times, with a maximum cutoff time of 300 seconds. In the tail suspension test, mice were individually placed in a square plastic chamber, with their tails (15 mm from the tip) suspended from a hook by a bandage. The test lasted for 10 minutes, and each mouse was tested only once. The total duration of immobility was measured. Trials were excluded if mice climbed their tails or fell during the test. For the open field test, mice were gently placed in the center of an open field (500×500×350 mm) and allowed to explore freely for 2 minutes. Their movement routes were recorded for the next 10 minutes. The total route distance and the number of center entries were analyzed for each mouse. The apparatus was cleaned with a 75% alcohol solution before and between tests to eliminate residual odors from other mice.

### Measurement of SOD activity, GSH/GSSG ratio and Fe^2+^ levels in tissues

Mice were sacrificed after behavior tests. Blood was cleared using ice-cold saline, and the substantia nigra pars compacta (SNc) tissues were rapidly extracted on ice and immediately frozen in liquid nitrogen. The tissues were then ground into powder. Superoxide dismutase (SOD) activities were measured using the Total Superoxide Dismutase Assay Kit with WST-8 (Beyotime, China). The GSH/GSSG ratio was determined with the GSH and GSSG Assay Kit (Beyotime, China). Ferrous ions (Fe^2+^) levels were detected using the Ferrous Iron Colorimetric Assay Kit (Elabscience, China).

### Immunohistochemistry (IHC) and immunocytochemistry (ICC)

Mice from the different groups were perfused with 4% paraformaldehyde in PBS for 20 min to fix the brains, followed by post-fixation in 4% PFA for 24 h. Brains were then dehydrated in a 20-30% sucrose gradient before being embedded in OCT compound. IHC were performed on 30 µm thick serial section including striatum and substantia nigra. Sections underwent antigen retrieval in EDTA solution (pH 9.0) for 20 minutes at 95°C and were washed 3 times in PBS. Blocking was performed for 1 hour in 10% goat serum with 0.25% Triton X-100 in PBS. Sections were then incubated overnight in primary antibodies solution. Following PBS washes, sections were incubated with secondary antibodies for 2 h, followed by washes in PBS. Finally, sections were flat-mounted on microscope slides and sealed with or without DAPI-containing mounting medium. For the in vitro study, cells were fixed with 4% paraformaldehyde in PBS for 10 min, followed by washes with PBS. Blocking was conducted for 1 hour using 10% goat serum with 0.1% Triton X-100 and 0.3 M glycine in PBS. Primary antibodies were applied and the cells were incubated overnight in PBS containing 10% goat serum and 0.3 M glycine. Following the overnight incubation with primary antibodies, cells were treated with secondary antibodies for 2 h. After washes with PBS, cells were mounted with a mounting medium, with or without DAPI. Details of Antibodies are listed in Table S2.

### Immunoblotting

Brain tissue samples (striatum and SNc) and cell preparations were lysed in RIPA buffer at 4°C for 30 minutes, followed by centrifugation at 12,000 g for 20 minutes. Protein concentrations were quantified using the BCA assay. Proteins were then diluted in sample loading buffer and denatured at 95°C for 10 min. Denatured protein samples were separated on 10% SDS-PAGE gels and transferred onto NC membrane (Pall, USA). The membranes were dried at room temperature for 1 h before being blocked with 5% nonfat milk in TBS. Primary antibodies were diluted in 5% BSA-TBST and incubated with the membranes overnight at 4°C on a shaker. After 3 washes in TBST, membranes were incubated with HRP-conjugated secondary antibodies for 2 h at room temperature. Following 3 times washes with TBST, protein bands were visualized using an Enhanced Chemiluminescence solution. Densitometry was measured by Image J Software (NIH).

### Whole-genome sequencing analysis

Total RNA was extracted from SNc using Total RNA Extraction Kit according to the protocol. Quality and integrity were determined using a Nanodrop spectrophotometer (Thermo Scientific, USA). Then, mRNA was purified from total RNA using poly-T oligo-attached magnetic beads. Fragmentation was carried out using divalent cations under elevated temperature in an Illumina proprietary fragmentation buffer. First-strand cDNA was synthesized using random oligonucleotides and Super Script II. Second-strand cDNA synthesis was subsequently performed using DNA Polymerase I and RNase H. Remaining overhangs were converted into blunt ends via exonuclease/polymerase activities and the enzymes were removed. After adenylation of the 3' ends of the DNA fragments, Illumina PE adapter oligonucleotides were ligated to prepare for hybridization. To select cDNA fragments of the preferred 400-500 bp in length, the library fragments were purified using the AMPure XP system (Beckman Coulter, USA). DNA fragments with ligated adaptor molecules on both ends were selectively enriched using Illumina PCR Primer Cocktail in a 15-cycle PCR reaction. Products were purified and quantified using the high-sensitivity DNA assay (Agilent, USA) on a Bioanalyzer 2100 system (Agilent, USA). The sequencing library was then sequenced on NovaSeq 6000 platform (Illumina, USA).

### Single-nucleus RNA sequencing meta-analysis

Public single-cell datasets (GSE178265, GSE157783 and GSE166790) were downloaded from the GEO database (https://www.ncbi.nlm.nih.gov/geo/). The GSE178265 dataset includes 37 non-PD samples and 34 PD samples. The GSE157783 dataset contains 6 non-PD samples and 5 PD samples. The GSE166790 dataset comprises 2 non-PD samples and 1 PD sample. Therefore, these datasets encompass a total of 45 non-PD samples and 40 PD samples, all derived from human substantia nigra pars compacta (SNc). Gene expression matrices were processed by R software (Version 4.3.1) with the Seurat package (Version 4.4.0 https://satijalab.org/seurat/). To identify subtypes in different states within microglia, we used a two round clustering strategy. Firstly, all cells were classified to seven cell types. Then, like we did on the all cells, dimensionality reduction and cell clustering were performed for microglia cells.

The expression matrices were transformed into a Seurat object using the CreateSeuratObject function. This was followed by data normalization, achieved through the NormalizeData and ScaleData functions, with the latter adjusting gene expression measurements to z-scores for standardization. The FindVariableFeatures function was utilized to identify the top 2,000 genes with the highest expression variability. For dimensionality reduction, Principal Component Analysis (PCA) was conducted on the normalized dataset utilizing the RunPCA function with default parameters. To remove batch effects, we employed the Harmony package (Version 1.2.0) using the top 20 principal components (PCs). The first round of analysis corrected for sample batch effects, while the second round addressed both sample and dataset batch effects 67. The integration of samples or datasets and visualization were facilitated through Uniform Manifold Approximation and Projection (UMAP). Cell type identification was informed by the expression profiles of known marker genes. Neurons were defined by the expression of the RBFOX3 (NeuN's coding gene), with DAergic neurons further identified through markers such as TH, SLC6A3, and SLC18A2. Endothelial cells were distinguished by CLDN5 and CDH5. MOBP and MOG are specifically expressed in oligodendrocytes, whereas OPCs show high expression of VCAN and PDGFRA. This differential expression allows for clear distinction between mature oligodendrocytes and OPCs. Microglia, the only resident immune cells of the brain, were identified by CD74, C1QB, and CX3CR1, whereas astrocytes were characterized by high expression of AQP4. Next, to explore the role of neuroinflammation in PD patients, we identified the microglia subpopulation, using the biomarker genes including, NAMPT, EGR1, IL1B, CCL2, CCL3, BAG3, HSPA4L, FKBP4, GPNMB, LPL, P2RY12, CACNB4, PCNXL2, OPRM1, ADGRG1, LRMDA, SHTN1, PRKN, FYB1, RPS3A and RPS23.

The integration of datasets and visualization were facilitated through Uniform Manifold Approximation and Projection (UMAP). Differentially gene expression analysis in each cluster was carried out using the FindAllMarkers function of the Seurat package, which employs a Wilcoxon rank-sum test to compare gene expression across clusters. Enrichment analysis of the differentially expressed genes (DEGs) was performed using the clusterProfiler package (Version 4.10.0), with gene ontology annotations sourced from org.Hs.eg.db (Version 3.18.0).

### Quantitative real-time PCR

Total RNA was extracted from either cells or tissue using a Total RNA Extraction Kit according to the manufacturer's instructions. The extracted RNA was immediately converted to cDNA using the cDNA Synthesis Kit. The relative expression of mRNAs in this study was determined using Taq Pro Universal SYBR qPCR Master Mix. All results were normalized to GAPDH gene expression. Quantitative analyses were conducted on a QuantStudio 1 Real-Time PCR System (Applied Biosystems, USA), following the manufacturer's guidelines. Primer sequences used in the study were listed in Table S3.

### Cell culture

Human pluripotent stem cells (hPSCs, hPSCs Line nciPS01, Nuwacell) were cultivated on Matrigel-coated, feeder-free culture dishes. Routine checks for mycoplasma were performed using a detection kit. hPSCs were maintained in ncTarget hPSC medium without antibiotics and passaged every 3-4 days by 0.5M EDTA. For experimental use, cells from passages 35-55 were utilized. Cells were cryopreserved using Mr. Frosty™ Freezing Container in cryopreservation medium for 8 h at -80°C before being stored in vapor-phase liquid nitrogen for long-term preservation. Differentiation into mesencephalic dopaminergic neurons followed the Kirkeby method with a biphasic WNT signaling activation strategy, as described in references 68, 69. Before differentiation, hPSCs were cultured onto laminin-521 with mTeSR plus medium without antibiotics for 3 passages. Then, colonies were dissociated into a single-cell suspension using Accutase. Floor-plate induction was carried out on laminin-111 coated dishes with a mixture of DMEM/F12 and Neurobasal containing N2 supplement, SHH-C24II (600 ng/ml), dual SMAD inhibitors (10 μM SB431542 and 100 ng/ml Noggin), and CHIR99021 (starting at 0.8 μM, increased to 8 μM on Day 4, and reduced to 3 μM on Day 10). From Day 11 onwards, the base medium was switched to Neurobasal containing B27 supplement, ascorbic acid (0.2 mM), dcAMP (500 µM), DAPT (1 µM), GDNF (10 ng/ml), BDNF (20 ng/ml), and TGFβ3 (1 ng/ml). DAergic neurons would mature and used for the experiment on Day 55. Additionally, a 2-step method was employed to generate midbrain DAergic neurons. Initially, hPSCs were induced to form high-purity neural progenitor cells (NPCs), which were subsequently differentiated into midbrain neurons, comprising approximately 92% β-tubulin III+ neurons (including about 12% TH^+^ DAergic neurons) and 8% astrocytes, using the SMADi Neural Induction kit and Midbrain Neuron Differentiation/Maturation kit.

The human neuroblastoma SH-SY5Y cell line was obtained from the Institute of Biophysics, Chinese Academy of Sciences. Cells were cultured in a 1:1 mixture of Minimum Essential Medium (MEM) and F12 Medium, supplemented with 10% fetal bovine serum (FBS), 1% MEM Non-Essential Amino Acids (NEAA), 1% sodium pyruvate, and 1% penicillin/streptomycin. Cultivation was conducted under a 5% CO_2_ atmosphere at 37°C. For gene editing in SH-SY5Y cells, we designed gRNAs using the CRISPR design tool available at http://crispor.tefor.net/. Subsequently, single-cell clones were isolated using flow cytometric cell sorting. We then employed Sanger sequencing to screen and identify the appropriate knockout cell clones.

### Cell viability and cell death measurement

Cell viability was assessed by MTS assay, conducted according to the manufacturer's instructions. Briefly, 20µl MTS solution was diluted in 100µl Hank's Balanced Salt Solution (HBSS) containing 5% FBS and added to each well. After incubating for 2 h at 37°C, absorbance was measured at 490 nm using microplate reader (Thermo Scientific, USA). Cell death was assessed by Calcein&PI staining coupled with microscopy or flow cytometry (Beckman Coulter, USA).

### Measurement of mitochondrial function, ferroptosis-related peroxidation and labile iron in vitro

JC-1 staining was utilized to measure mitochondrial membrane potential. In brief, the cells were stained 10 µM JC-1 after incubation for 20 min at 37 °C. The activity of mitochondrial Complex I were determined using Mitochondrial Complex I assay kit (Abcam, USA).

2',7'-Dichlorofluorescin diacetate (H_2_DCFH-DA) and MitoSOX were used to evaluate intracellular ROS and mitochondrial ROS content, respectively. Cells were stained with 0.5 µM H_2_DCFH-DA after incubation for 10 minutes at 37°C; cells were incubated with 5 µM MitoSOX in serum-free medium for 30 minutes at 37°C and then analyzed by the same instruments. Subsequent detections were performed using microplate reader, flow cytometry or confocal microscope.

Lipid peroxidation in cells and mitochondria was measured using C11-BODIPY and MitoPeDPP according to the manufacturer's instructions. Cells were incubated with 10 µM C11-BODIPY in serum-free medium for 30 minutes at 37°C and then analyzed by the same instruments. Cells were incubated with 0.5 µM MitoPeDPP for 15 minutes at 37°C, washed twice post-harvesting, and analyzed for lipid peroxidation levels using either a microplate reader, flow cytometry, or confocal microscope.

Lipid peroxidation was measured in vitro using C11-BODIPY according to manufacturer's instructions; cells were incubated with 10 µM C11-BODIPY in serum-free medium for 30 minutes at 37°C and then analyzed by the same instruments. For mitochondrial lipid peroxidation, we used MitoPeDPP, a probe designed to target mitochondrial lipids. Cells were incubated with 0.5 µM MitoPeDPP for 15 minutes at 37°C, washed twice post-harvesting, and analyzed for lipid peroxidation levels using either microplate reader, flow cytometry, or confocal microscope.

Intracellular Fe^2+^ levels were quantified using FerroOrange. Cells were incubated with 1 µM FerroOrange for 15 minutes at 37°C. The fluorescent signals were recorded with a microplate reader and visualized by live-cell confocal microscope.

### Dual luciferase reporter assays

To construct the luciferase reporter vectors, Hspb1 promoter sequences (3046 bp) or its 3 fragments (1-857; 858-1958; 1959-3046) were subcloned into the pGL4.10-basic luciferase vector. SH-SY5Y cells were plated in 96-well plates and allowed to attach overnight. Subsequently, each promoter-reporter construct was introduced into the cells using Lipofectamine 2000. Co-transfection with a renilla plasmid served as a control for transfection efficiency. The baseline luciferase activity was assessed using the pGL4.10 vector alone. After a 24-hour transfection period, cells were treated with MPP^+^ and CFA for an additional 24 hours. Cell lysates were then prepared, and luciferase activity was quantified using a microplate reader.

### Chromatin immunoprecipitation (ChIP) assay

Chromatin immunoprecipitation was performed using the ChIP Kit (Abclonal) following the manufacturer's protocol. Briefly, for each group, 1.5 × 10^7^ DAergic neurons were cross-linked with 1% formaldehyde for 15 min at room temperature. The cross-linking was then quenched with glycine stop solution. The chromatin was sonicated in lysis buffer to 200-1000 bp fragments. Immunoprecipitation was carried out with rabbit anti-Nrf2 or control rabbit anti-IgG antibodies. Immunoprecipitated DNA was then quantified via qPCR using primers specific for the Hspb1 promoter region (Forward primer 5'-AGTTAGATTCTTTGTGCC-3'; Reverse primer 5'-ACCTTCATATTTGCTGT CCCTT-3').

### Statistics and reproducibility

All data were presented as the mean ± SEM. All statistical analyses were performed using GraphPad Prism version 9.0. The statistical significance was assessed using t test or one-way analysis of variance (ANOVA) with TUKEY post hoc tests. Sample sizes are indicated in each figure legend, and statistically significant results are denoted in the corresponding figures and their legends. For in vitro experiments, including experiments using microplate reader, each assay was performed in 3 replicates to ensure reproducibility and minimize variability.

## Results

### snRNA-seq data from PD patients indicates upregulation of Nrf2 and Hspb1, highlighting the key role of ferroptosis in PD progression

Compared to other neurodegenerative diseases, PD is particularly linked to cell death. Thus, understanding the key signaling pathways that regulate cell death in PD is essential to arrest its pathological progression. The role of ferroptosis in PD has recently come under scrutiny. However, definitive evidence detailing the influence of ferroptosis in PD patients and identifying the molecules that regulate this type of cell death remains elusive.

**Figure 1:**
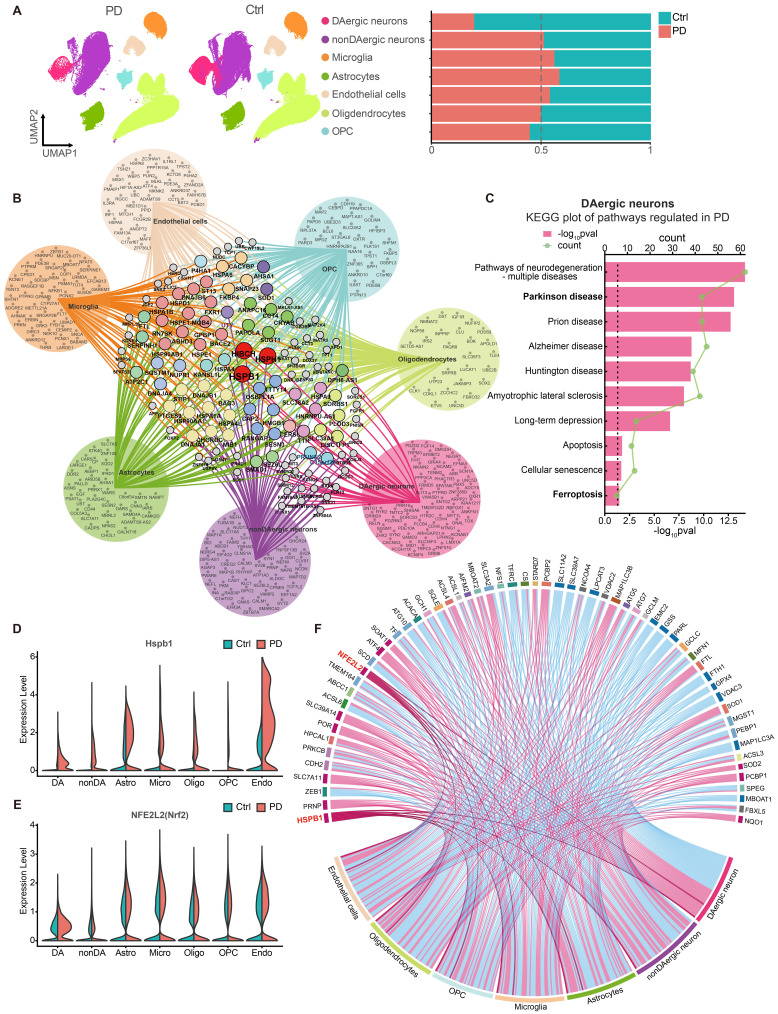
** The Upregulation of Nrf2 and Hspb1 serve as the Core Molecules in the Pathological Mechanisms of PD. A** Uniform Manifold Approximation and Projection (UMAP) embeddings of human midbrain nuclei from PD patients (PD) and healthy controls (Ctrl). The nuclei were annotated into dopaminergic neurons (DAergic neurons), non-dopaminergic neurons (nonDAergic neurons), Microglia, Astrocytes, Endothelial cells (including pericytes), Oligodendrocytes, Oligodendrocyte precursor cells (OPCs), based on biomarker genes. The proportion of each cell type in PD and Ctrl group were presented. **B** Network of the top 100 upregulated coding DEGs across different cell types in PD compared to Ctrl group. Genes uniquely upregulated in one single cell type are positioned within the circle, while genes expressed in two or more cell types are located in the network's center, connected by lines to their respective cell types. **C** GO analysis of DEGs in DAergic neurons identified significant terms regulated in PD. **D-E** Changes of Hspb1 and Nfe2l2 mRNA level in each cell type in PD and Ctrl. **F** Changes of ferroptosis-related genes among various cell types in PD were shown in the chord diagram. Red connections represent significant upregulation, while blue represent significant downregulation.

To investigate whether ferroptosis is involved in the pathological progression of PD patients, we integrated three snRNA-seq datasets (GSE178265, GSE157783, and GSE166790) from GEO database and conducted data analysis. The details of the dataset analysis are described in the Methods section. We classified cells based on established biomarkers, identifying seven distinct cell populations (Figure S1A-S1B). Comparisons between PD patients and healthy controls revealed a significant decline in dopaminergic (DAergic) neurons in PD patients, with corresponding increases in astrocytes and microglia (Figure 1A). From each cell type, we extracted the top 100 upregulated DEGs (differentially expressed genes) in PD (p < 0.001, fold change >0.3, sorted by fold change in descending order), to construct gene networks and pinpoint genes commonly upregulated across all cell types. Notably, Hspb1, Hsph1, and Hibch were consistently upregulated in each cell type (Figure 1B), highlighting the crucial role of Hspb1 in PD. Specifically, HIBCH (3-Hydroxyisobutyryl-CoA Hydrolase) is crucial in valine degradation, linked to mitochondrial homeostasis and neuronal health 70, 71, yet its specific function in PD is still to be explored. HSPH1, a heat shock protein, serves as a molecular chaperone, facilitating protein folding and stabilization under stress, and has been observed to stabilize slc7a11, suggesting a potential role in ferroptosis inhibition 72. The exact contributions of HSPH1 to PD, however, remain unclear. Additionally, in DAergic neurons and astrocytes affected by PD, DEGs were significantly enriched in pathways pertinent to various neurodegenerative diseases, apoptosis and ferroptosis (Figure 1C and Figure S1C). This pattern suggests that the loss of DAergic neurons may involve both apoptosis and ferroptosis, supporting the insights gained from RNA-seq analyses.

To further explore the regulation of ferroptosis-related genes in different cell types of PD patients, we compiled a ferroptosis gene set from the ferroptosis datasets of AmiGO2 and KEGG, as well as from ferroptosis genes mentioned in two review articles 73, 74. We documented the significant upregulation and downregulation of these genes across different cell types (Figure 1F). Remarkably, Hspb1 emerged as the gene most consistently upregulated across all cell types (Figure 1D, 1F). Meanwhile, NFE2L2 (Nrf2) also exhibited substantial upregulation in all cell types except for non-DAergic neurons, where its expression difference was not significant (Figure 1E-1F). In addition, in PD patients, SLC7A11 was upregulated across all cell types, whereas downregulation of GPX4 was observed in cells other than astrocytes and microglia (Figure 1F). The role of Nrf2 in regulating ferroptosis is controversial. Some studies argue that Nrf2 does not play a significant role in regulating ferroptosis, at least in RSL3-induced ferroptosis 75. However, other research supports the notion that Nrf2 can transcriptionally upregulate genes such as SLC7A11, imparting anti-ferroptosis effects 76. Considering the broad upregulation of Nrf2 across cell types in PD patients, our study focused on the implications of Nrf2 in modulating cell death in PD.

### CFA abrogates motor deficits and prevents dopaminergic neuron loss in PD mouse model

Nrf2 is tightly regulated under normal physiological conditions, confined to the cytoplasm by the Keap1-Nedd8-Cul3-Rbx1 complex and rapidly degraded by the ubiquitin-proteasome pathway 77. In exploring Nrf2 activation, key strategies include: either knocking out or suppressing Keap1 expression to decrease Nrf2 ubiquitination rates, or using specific Nrf2 activators that may either oxidize Keap1 78 or cause the accumulation of proteins like p62, DPP3, WTX, CDK20, and p21, which interfere with the Keap1 complex 79, 80. Both approaches have their advantages and disadvantages; knocking out Keap1 may inadvertently impinge upon its other physiological functions 81, and Nrf2 activators often struggle with selectivity issues 82. Furthermore, the effectiveness of many Nrf2 activators is greatly reduced in the brain due to the blood-brain barrier. Recently, a novel aldehyde-based Nrf2 agonist, Coniferaldehyde (CFA), has shown promise in overcoming this barrier and effectively activating Nrf2 83. Consequently, we employed CFA to activate Nrf2 in the in vivo PD model.

To assess the neuroprotective potential of CFA, we first evaluated its effects in MPTP-induced mouse model of PD 62. Following an evaluation of various concentrations, we determined that a 14-day regimen of 10 mg/kg CFA was optimal in improving balance impairments and reducing the loss of dopaminergic neurons in the substantia nigra pars compacta (SNc), as indicated by the pole test (Figure S2A) and immunoblot analysis of TH levels (Figure S2B). This dose was thus chosen for subsequent analyses. Further behavioral assessments and evaluations of SNc dopaminergic neuron viability revealed that CFA significantly ameliorated MPTP-induced motor deficits. This improvement was evident in wire hanging test (Figure S2C), and tail suspension test (Figure S2D). Moreover, depressive-like motor deficits were mitigated, as shown by increased central area entries and total distance traveled in the open field test (Figure S2E-2G). Immunoblot analysis confirmed that CFA restored TH protein levels in the SNc and striatum (Figure S2H-2J).

**Figure 2:**
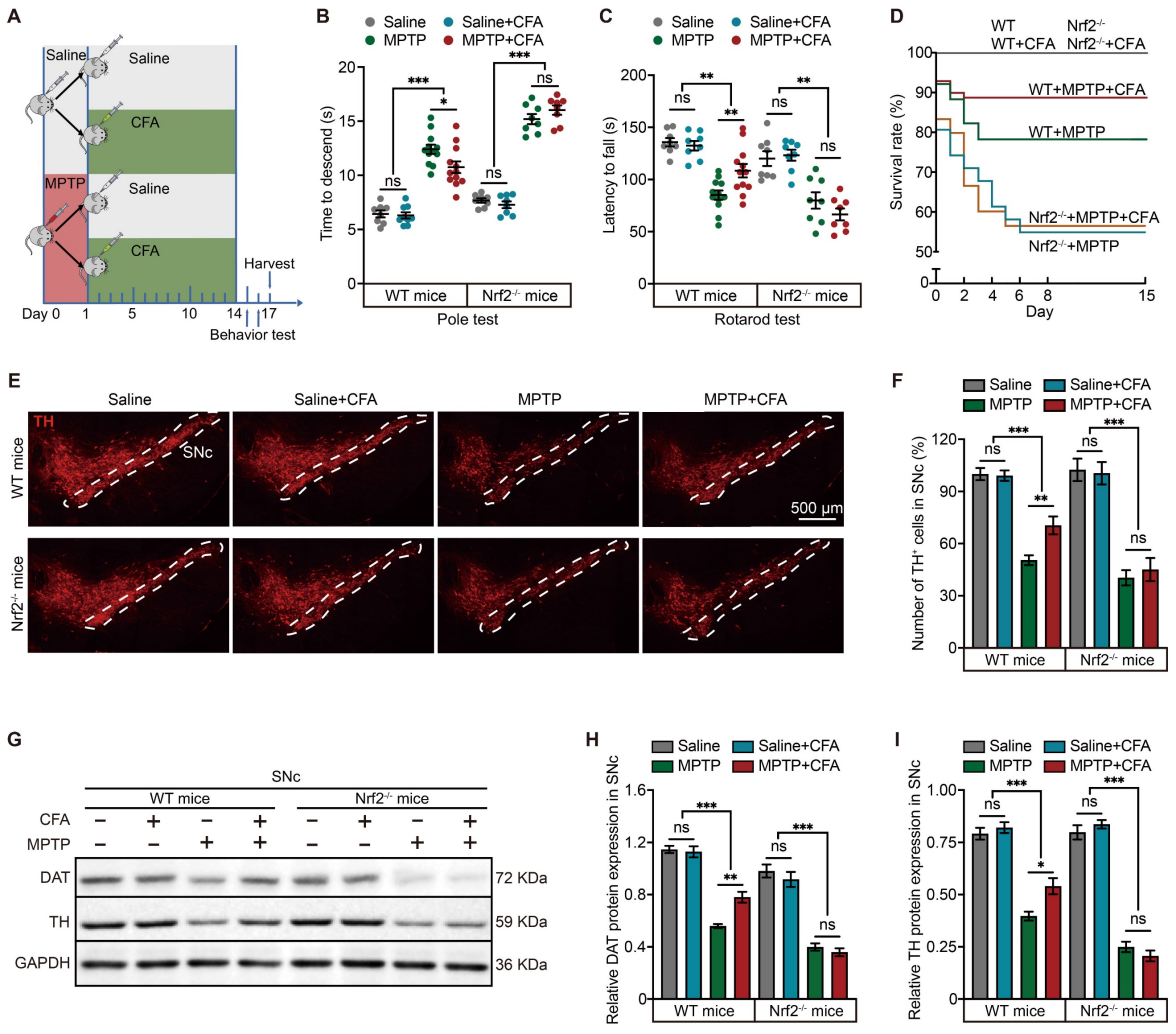
** Nrf2 Activation Prevents Neuronal Death and Mitigates Motor Deficits in MPTP-Induced Mouse PD Model. A** Schematic of the experimental paradigm for the administration of MPTP and CFA. Eight-week-old C57BL/6 mice were administered intraperitoneally with MPTP (20 mg/kg) or saline (as a vehicle) four times at 2 h intervals on the first day. Subsequently, mice were treated with CFA or dimethyl sulfoxide (DMSO as a vehicle) daily for 14 days. After the behavioral tests, brain tissues were harvested for molecular analysis. **B-C** Pole test and rotarod test were conducted to assess the protective effects of CFA on motor function in WT and Nrf2^-/-^ mice. CFA administration ameliorated MPTP-induced motor deficits in WT mice, while no significant improvement was observed in Nrf2^-/-^ mice. Data are presented as mean ± SEM. Statistical analyses were performed using t test. *p < 0.05, **p < 0.01, ***p < 0.001, ns, not significant (n = 8-12 mice per group). **D** Mouse survival rates were recorded in this experiment (n = 12 mice per group). **E-F** Representative immunofluorescence images of TH staining in SNc. The thickness of the brain sections is 30 µm. Scale bars are as indicated in the images. Stereological counts of TH^+^ nigral neurons. Data are presented as mean ± SEM. Statistical analyses were performed using t test. **p < 0.01, ***p < 0.001, ns, not significant (n = 5-14 mice per group). **G-I** Representative Immunoblotting images for DAT and TH in SNc lysates from the mice. Quantification of DAT and TH protein levels in SNc normalized to GAPDH. Data are presented as mean ± SEM. Statistical analyses were performed using one-way ANOVA followed by TUKEY post hoc tests. **p < 0.01, ***p < 0.001, ns, not significant (n = 4 mice per group).

Considering the potential lack of specificity in Nrf2 inducers, we further explored whether the therapeutic effects of CFA were mediated through Nrf2 activation. For this purpose, Nrf2 knockout (Nrf2^-/-^) mice were utilized in this study, as illustrated in the diagram (Figure 2A). Behavior tests, specifically the pole test and rotarod test, were conducted, revealing that the absence of Nrf2 partially reduced the protective effects of CFA (Figure 2B-2C). Additionally, while CFA significantly improved survival rates in WT mice, this enhancement was not observed in Nrf2^-/-^ mice (Figure 2D), highlighting the role of Nrf2 in mediating the beneficial effects of CFA. It is important to note that under normal physiological conditions, Nrf2^-/-^ mice displayed similar motor coordination and reproductive capabilities as WT mice; however, their resistance to toxic stress was notably diminished due to Nrf2 deletion. We further assessed the protective effects of CFA on dopaminergic neurons in the SNc both mouse types. Tyrosine hydroxylase (TH), a rate-limiting enzyme in the synthesis of dopamine, and dopamine transporter (DAT) are used to quantify the amount and quality of dopaminergic neurons. We counted the neurons by the positive staining for TH (the area within the white dashed lines represents the SNc region). The results indicated that CFA inhibited dopaminergic neuron loss (Figure 2E, 2F) and maintained DAT and TH protein levels (Figure 2G-2I). In contrast, these protective effects were absent in Nrf2^-/-^ mice, confirming the cytoprotective effect on preventing neuronal death is dependent on Nrf2 activation.

### The anti-ferroptosis effect of Nrf2 activation is potentially mediated by Hspb1 upregulation

**Figure 3:**
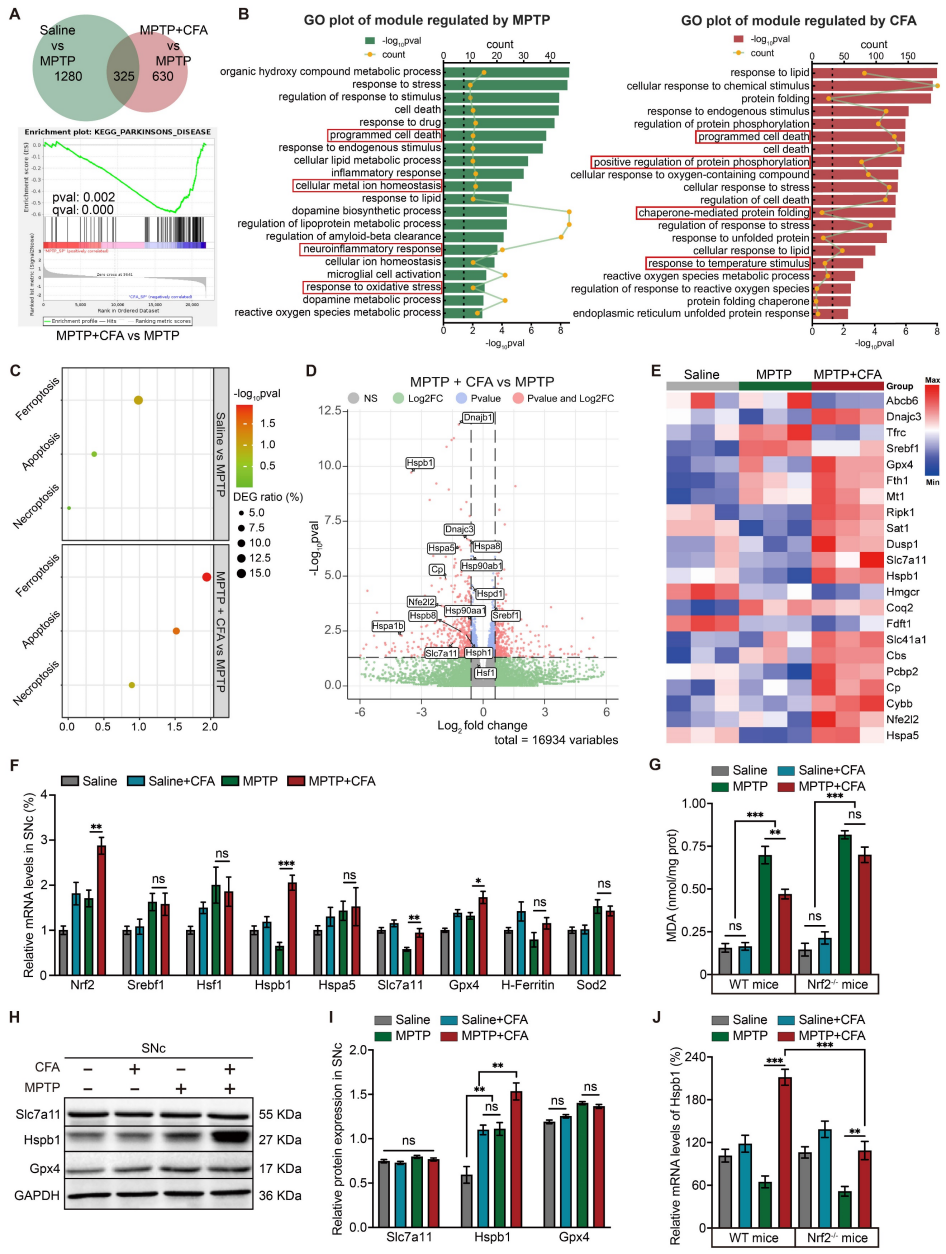
** RNA-seq Revealed Relationship between Nrf2-Upregulated and MPTP-Induced Genes with Ferroptosis and Highlighted Hspb1's Potential Role in Cell Death Pathways.** To explore the gene alterations induced by MPTP and CFA, total mRNA samples from the SNc of mice were collected at day 7 for RNA sequencing. **A** Venn diagram illustrates the number of significantly changed genes in each pairwise comparison (Saline vs. MPTP, and MPTP+CFA vs. MPTP) with a fold change threshold of ±1.2 and p-value < 0.05. Gene set enrichment analysis (GSEA) identified KEGG_PARKINSONS_DISEASE-related gene differences between the MPTP+CFA and MPTP groups (FWER p = 0.002, FDR q < 0.001). **B** Gene ontology (GO) analysis of the significant terms regulated by MPTP (left) and CFA (right), with key terms of interest highlighted in red boxes. **C** KEGG analysis highlighted several significant programmed cell death pathways regulated by MPTP and CFA. **D** Volcano plots of DEGs upregulated by CFA. Genes with fold change > 1.2 and p < 0.05 are shown in red. Some notable ferroptosis-related genes are noted. **E** Heatmap of ferroptosis-related DEGs of MPTP induced PD mice with or without CFA administration. **F** Relative mRNA levels of the ferroptosis-related genes and antioxidant genes in SNc from the mice were measured using qPCR. Data are presented as mean ± SEM. Statistics were assessed using t test. *p < 0.05, **p < 0.01, ***p < 0.001, ns, not significant (n = 5 mice per group). **G** Quantification of MDA levels in SNc lysates from the WT and Nrf2^-/-^ mice. The results showed CFA reduced lipid peroxidation levels in WT mice, but not in Nrf2^-/-^ mice. Data are presented as mean ± SEM. Statistics were assessed using t test. **p < 0.01, ***p < 0.001, ns, not significant (n = 5 mice per group). **H-I** Representative Immunoblotting images and quantification of Slc7a11, Gpx4 and Hspb1 in SNc lysates of the mice in different groups. Data are presented as mean ± SEM. Statistics were assessed using t test (n = 5 mice per group). **J** Relative mRNA expression of Hspb1 in the SNc from WT and Nrf2^-/-^ mice was quantified with or without CFA administration once daily for 7 days after MPTP injection. Data are presented as mean ± SEM. Statistics were assessed using one-way ANOVA followed by TUKEY post hoc tests. **p < 0.01, ***p < 0.001 (n =5 mice per group).

The protective effects of Nrf2 activation against MPTP-induced death of DAergic neurons led us to investigate the downstream mechanisms mediated by CFA. RNA sequencing (RNA-seq) analysis of SNc from three groups (Saline, MPTP, MPTP+CFA) showed 315 genes were affected by MPTP administration, whereas 616 genes were reversed by CFA administration, as illustrated in the Venn diagram (Figure 3A). CFA restored the PD-related genes decreased by MPTP, including TH, and SLC6A3, as identified through gene set enrichment analysis (GSEA) (Figure 3B). Gene Ontology (GO) analysis highlighted that exposure to MPTP significantly triggered pathways related to programmed cell death, with a particular increase in ferroptosis compared to apoptosis and necroptosis, according to Kyoto Encyclopedia of Genes and Genomes (KEGG) pathway analysis (Figures 3C). Additionally, the data indicated that CFA's cytoprotective effects are associated with the modulation of cell death pathways, particularly through a significant influence on ferroptosis signaling pathways. Several genes that counteract ferroptosis, such as Gpx4, Slc7a11, and Hspb1, were upregulated following CFA administration (Figure 3D-3E).

Ferroptosis was first identified during investigations into the anti-tumor agent Erastin, which disrupts mitochondrial homeostasis and induces non-apoptotic cell death, a mechanism similar to that observed with MPTP in this study 84, 85. Erastin acts by inhibiting the cystine-glutamate antiporter, System Xc-, leading to cystine deprivation and consequent iron-independent ferroptosis 86. To verify CFA's capacity to counteract ferroptosis, we established an in vitro model using Erastin and two other well-known ferroptosis inducers, RSL3 and FIN56. RSL3 binds covalently to GPX4, inhibiting it, while FIN56 promotes GPX4 degradation and activates squalene synthase, reducing cellular resistance to ferroptosis 87, 88. Exposing SH-SY5Y neuroblastoma cells with Erastin (10 μM), RSL3 (10 μM), and FIN56 (1 μM) for 24 hours induced ferroptosis, whereas co-treatment with CFA (100 μM) significantly reduced intracellular lipid peroxidation and cell death (Figure S3A-S3C), indicating that CFA's protective mechanisms may extend beyond SLC7A11 and GPX4 modulation.

The hallmark of ferroptosis is the accumulation of intracellular lipid peroxides. Malondialdehyde (MDA) serves as a crucial biomarker for lipid peroxidation 89. In vivo, MPTP increased MDA level in the SNc of mice, a process that CFA could mitigate, an effect not observed in Nrf2^-/-^ mice, underlining Nrf2's crucial role in mediating CFA's anti-ferroptosis actions (Figure 3G). Considering mitochondrial toxic effect of MPTP, we also measured intracellular SOD activity and Sod2 mRNA levels 90. While Sod2 mRNA changes were not significant (Figure 3F), SOD activity increased post-CFA treatment (Figure S3D). Furthermore, CFA restored GSH/GSSG ratios (Figure S3E). RNA-seq data showed changes in ion homeostasis-related genes after MPTP exposure, hinting at a role for ion imbalance in cell death. Specifically, MPTP impairs mitochondrial oxidative phosphorylation, reducing mitochondrial membrane potential and exacerbating mitochondrial damage, a primary source of Fe^2+^ and ROS 91. Alterations in other cellular ions, including increased Ca^2+^ and decreased Mg^2+^, further promote cell death and decrease mitochondrial resistance to oxidative stress, facilitating Fe^2+^ entry and triggering the Fenton reaction 92-94. However, studies indicate that MPTP/MPP^+^ induces lipid peroxidation only at non-physiological Fe^2+^ concentrations 95. Therefore, elevated Fe^2+^ may be essential for triggering MPTP-induced ferroptosis. Through differential centrifugation, we removed the two primary Fe^2+^ pools, the mitochondria and cell nuclei, and measured the Fe^2+^ in the cytosol. The results showed that MPTP increased cytosolic Fe^2+^, which was not mitigated by CFA (Figure S3F). These results suggest that CFA's inhibition of ferroptosis may primarily enhance antioxidant enzyme activity and GSH synthesis efficiency rather than controlling Fe^2+^ levels to reduce Fenton reaction outcomes. Further research is needed to assess if activating Nrf2 could ameliorate mitochondrial dysfunction.

To assess which gene plays a crucial role in mitigating lipid peroxidation, we quantified the mRNA level of selected anti-ferroptotic genes including H-Ferritin, Sod2, SLC7A11, Hspb1 and Hspa5 using quantitative PCR (qPCR). Notably, mRNA levels of GPX4, SLC7A11, and Hspb1 were significantly modulated by CFA (Figure 3F). However, protein levels of GPX4 and SLC7A11 did not show significant changes across experimental groups, whereas Hspb1 protein expression was substantially elevated following CFA administration (Figure 3H-3I). This suggested that the anti-ferroptotic effect of CFA may be closely associated with the upregulation of Hspb1. Currently, the function of Hspb1 in inhibiting ferroptosis is not completely understood; however, it is known that Hspb1 overexpression can diminish cellular death and oxidative stress 96. In PD, it is generally believed that Hspb1 helps prevent the accumulation of α-synuclein, thereby providing a cytoprotective effect 97. RNA-seq analysis led to further exploration of transcription factors that might influence Hspb1 expression. Although Hspb1 upregulation is typically linked to Hsf1, a primary transcription factor for many HSPs 98, in the MPTP model of PD, the increase in Hspb1 did not appear to be regulated by Hsf1 or Srebf1, the latter also being considered a potential anti-ferroptosis transcription factor (Figure 3F) 99. Moreover, while CFA significantly increased Hspb1 levels in Nrf2-deficient (Nrf2^-/-^) mice compared to untreated MPTP groups, the extent of this increase was markedly less than that observed in WT mice (Figure 3J), indicating that Nrf2 activation plays a critical role in Hspb1 upregulation in PD.

### CFA mitigates MPP^+^-induced ferroptosis in hPSC-derived dopaminergic neurons by activating Nrf2 and enhancing Hspb1 expression

**Figure 4:**
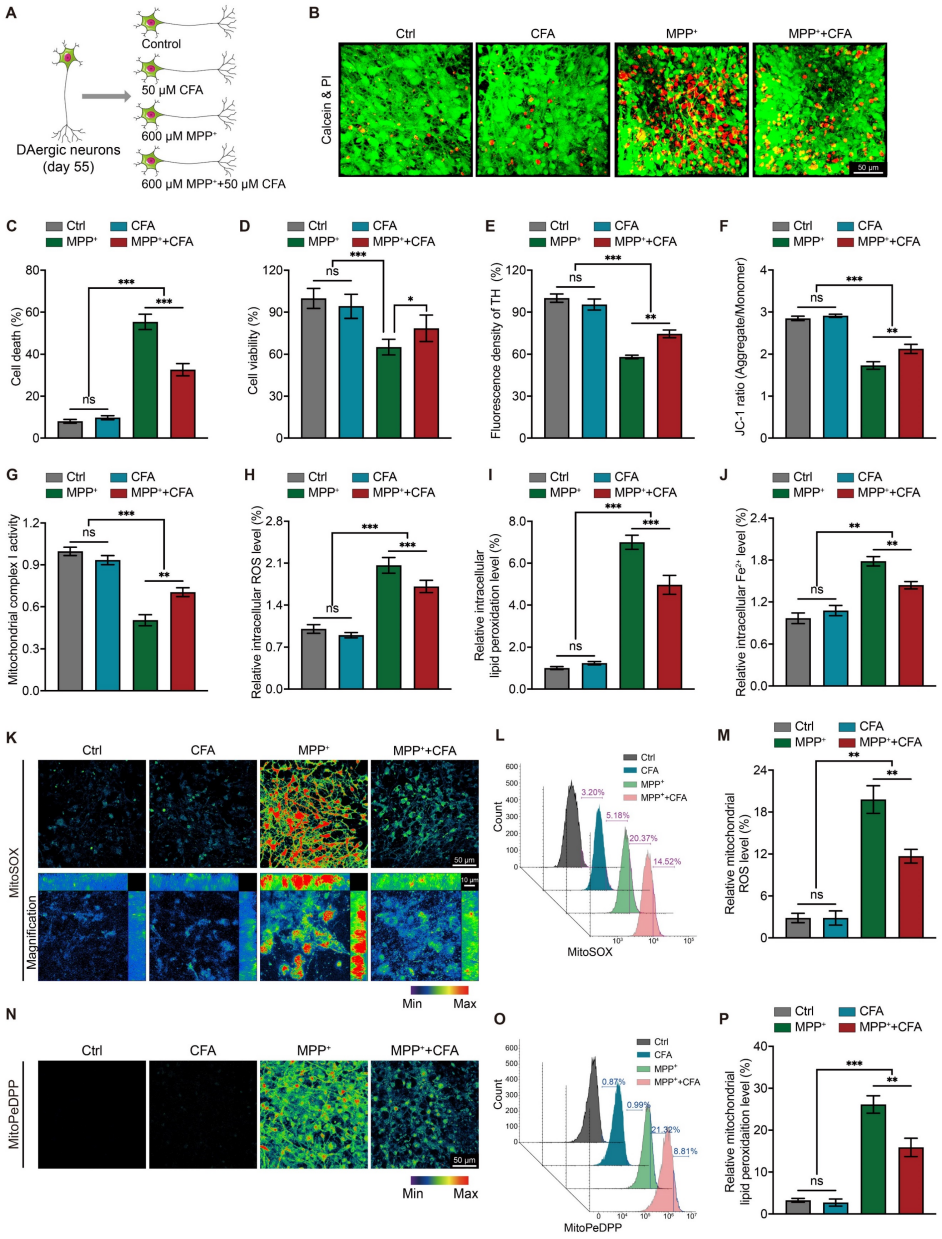
** CFA prevents MPP^+^-induced cell death and mitochondrial dysfunction and upregulates Hspb1 in hPSC-derived dopaminergic neurons. A** Diagrams of the experimental design and the details of experiments. The differentiation strategy employed for generating DAergic neurons was shown in Figure S4. **B** Representative images of Calcein and PI staining of DAergic neurons. Live cells were stained by Calcein, which appeared green. Meanwhile PI staining was employed to detect dead cells, which appeared red. Scale bar, 50μm. **C** Quantification of cell death. Data are presented as mean ± SEM. Statistics were assessed using one-way ANOVA followed by TUKEY post hoc tests. *p < 0.05, ***p < 0.001, ns, not significant (n = 6). **D** Cell viability was quantified by MTS assay. Data are presented as mean ± SEM. Statistics were assessed using one-way ANOVA followed by TUKEY post hoc tests. ***p < 0.001, ns, not significant (n = 6). **E** TH^+^ fluorescent density of DAergic neurons is quantified. Data are presented as mean ± SEM. Statistics were assessed using one-way ANOVA followed by TUKEY post hoc tests. **p < 0.01, ***p < 0.001, ns, not significant (n = 6). **F** Fluorescence of aggregate/monomer was detected using a fluorescence plate reader. The JC-1 ratio was determined by calculating the ratio of fluorescence intensity emitted by the aggregates (525/590 ± 15 nm) to the monomers (488/530 ± 15 nm). Statistics were assessed using one-way ANOVA followed by TUKEY post hoc tests. Data are presented as mean ± SEM. **p < 0.01, ***p < 0.001, ns, not significant (n = 8). **G** Mitochondria were isolated and the concentration of mitochondrial protein from different groups were normalized. Then mitochondrial Complex I enzyme activity were measured using colorimetric assay kit. Data are presented as mean ± SEM. Statistics were assessed using one-way ANOVA followed by TUKEY post hoc tests. **p < 0.01, ***p < 0.001, ns, not significant (n = 6). **H-J** Intracellular ROS, lipid peroxidation and Fe^2+^ levels in neurons were measured. Data are presented as mean ± SEM. Statistics were assessed using one-way ANOVA followed by TUKEY post hoc tests. **p < 0.01, ***p < 0.001, ns, not significant (n = 6). **K** Representative 2D and magnified 3D images of mitochondrial ROS levels using MitoSOX staining (510/580 ± 20 nm). Scale bar, 50μm and 10μm. **L-M** After stained by MitoSOX, DAergic neurons from different groups were detected by flow cytometry for quantification of mitochondrial ROS levels. Data are presented as mean ± SEM. Statistics were assessed using one-way ANOVA followed by TUKEY post hoc tests. **p < 0.01, ns, not significant (n = 4). **N** Representative images of mitochondrial lipid peroxidation levels using MitoPeDPP staining (488/535 ± 30 nm). Scale bar, 50μm. **O-P** Quantification of mitochondrial lipid peroxidation levels by flow cytometry. Data are presented as mean ± SEM. Statistics were assessed using one-way ANOVA followed by TUKEY post hoc tests. **p < 0.01, ***p < 0.001, ns, not significant (n = 4).

To explore how MPTP induces ferroptosis and assess the protective role of Nrf2 activation, we differentiated human pluripotent stem cells (hPSCs) into high-purity mesencephalic dopaminergic neurons (DAergic neurons) based on the Kirkeby method 68, 69. This differentiation resulted in approximately 86.13% TH^+^ neurons, with negligible astrocyte (GFAP^+^) contamination (Figure S4A-S4C). We then exposed these DAergic neurons to 600 μM MPP^+^ with or without 50 μM CFA for 36 hours and evaluated CFA's cytoprotective effects (Figure 4A). Similar to our observations in the MPTP mouse PD model, exposure to MPP^+^ for 36 hours resulted in over 50% cell death, while co-treatment with CFA significantly reduced cell death (Figure 4B-4C), and enhanced cell viability (Figure 4D). Immunocytochemistry (ICC) results showed that CFA treatment preserved TH protein levels and maintained the structural integrity of neuronal soma and axons (Figure 4E and Figure S5A). Considering that MPTP/MPP^+^ impairs mitochondrial complex I 100, we evaluated mitochondrial membrane potential (ΔΨm) using JC-1 staining (Figure 4F and Figure S5B) and measured complex I activity (Figure 4G). Results indicated that MPP^+^ exposure resulted in a 50% reduction in mitochondrial membrane potential, which was ameliorated by CFA treatment. Additionally, CFA effectively reduced ROS and lipid peroxidation levels induced by MPP^+^ (Figure 4H-4I and Figure S5C-S5D). We also measured total Fe^2+^ levels in DAergic neurons, observing that Nrf2 activation by CFA reduced abnormal intracellular Fe^2+^ levels caused by MPP^+^ (Figure 4J and Figure S5E). To verify whether CFA alleviated mitochondrial Fe^2+^ levels. Further experiments using MitoSOX and MitoPeDPP probes demonstrated that CFA markedly protected mitochondria from ROS and lipid peroxidation (Figure 4K-4P), surpassing the general improvement seen in intracellular ROS and lipid peroxidation.

To investigate whether CFA's protection against MPP^+^ induced cell death is mediated through Nrf2 activation and Hspb1 upregulation, we analyzed Nrf2 nuclear translocation. Nrf2 translocation to the nucleus was significantly elevated following treatment with MPP^+^ or CFA compared to the control group, with the highest increase observed under combined CFA and MPP^+^ treatment (Figure S6A and S6C). RNA-seq data suggested that CFA's cytoprotective effects might be linked to protein phosphorylation, and previous studies have shown that phosphorylated Hspb1 enhances proteostasis and prevents cell death 96, 101. We therefore monitored both total and phosphorylated Hspb1 (p-Hspb1).

We found a notable increase of Hspb1 protein levels in the cytoplasm after incubated with CFA and MPP^+^, alongside detectable Hspb1 within the nucleus (Figure S6B and S6D). To quantify Hspb1 in different cellular compartments, we fractionated nuclear proteins and found a significant increase in Hspb1 in both the nuclear and cytoplasmic fractions following CFA administration (Figure S6F). Furthermore, we observed a marked increase in cytoplasmic p-Hspb1 after MPP^+^ exposure, which was significantly reduced when co-incubated with CFA (Figure S6E). Co-labeling of p-Hspb1 with DAPI indicated that p-Hspb1 is predominantly cytoplasmic, unlike the non-phosphorylated Hspb1 (Figure S6B), suggesting a need for further investigation into the specific roles of p-Hspb1.

We also employed SH-SY5Y cells for in-vitro study as they possess similar characteristics to dopaminergic neurons, also express DAT, and can uptake MPP^+^. Similar to DAergic neurons, in SH-SY5Y cells, exposure to MPP^+^ or CFA resulted in the upregulation of Hspb1 protein levels in the cytoplasm (Figure S6G, S6H). Additionally, the nuclear localization of Hspb1 was enhanced when cells were treated with either MPP^+^ or CFA (Figure S6I). However, the changes in cytoplasmic p-Hspb1 were not substantial (Figure S6G, S6J). Interestingly, in SH-SY5Y cells, p-Hspb1 showed noticeable nuclear expression, and cells exposed to MPP^+^ exhibited a significant nuclear signal of p-Hspb1 (Figure S6G, S6K, highlighted by arrows). This suggests that p-Hspb1 may translocate to the nucleus to fulfill specific roles under stress conditions induced by MPP^+^. In contrast, following treatment with both MPP^+^ and CFA, only a subset of cells showed this marked nuclear expression of p-Hspb1, and interestingly, these cells typically displayed decreased total Hspb1 levels (Figure S6G, S6K).

Collectively, under normal physiological conditions, the expression of p-Hsp1 maintains at a low level in neurons and only be upregulated after exposure to MPP^+^, suggesting that the regulation of p-Hspb1 may not be directly related to Nrf2 activation. Furthermore, differences in the expression and localization of p-Hspb1 between neurons and SH-SY5Y cells are evident. p-Hspb1 exists in the nuclei of SY5Y cells, was upregulated under MPP^+^ exposure, suggesting a potential link to cellular responses to mitochondrial dysregulation-induced stress. Notably, the absence of nuclear p-Hspb1 in differentiated DAergic neurons implies that its functions could be restricted to proliferative tumor cells rather than normal neurons. This study focused primarily on observing changes in Hspb1 expression and its association with Nrf2 activation and cell death, without exploring the specific roles of nuclear p-Hspb1 in detail.

### CFA upregulates Hspb1 expression by enhancing the transcriptional efficiency of Nrf2

**Figure 5:**
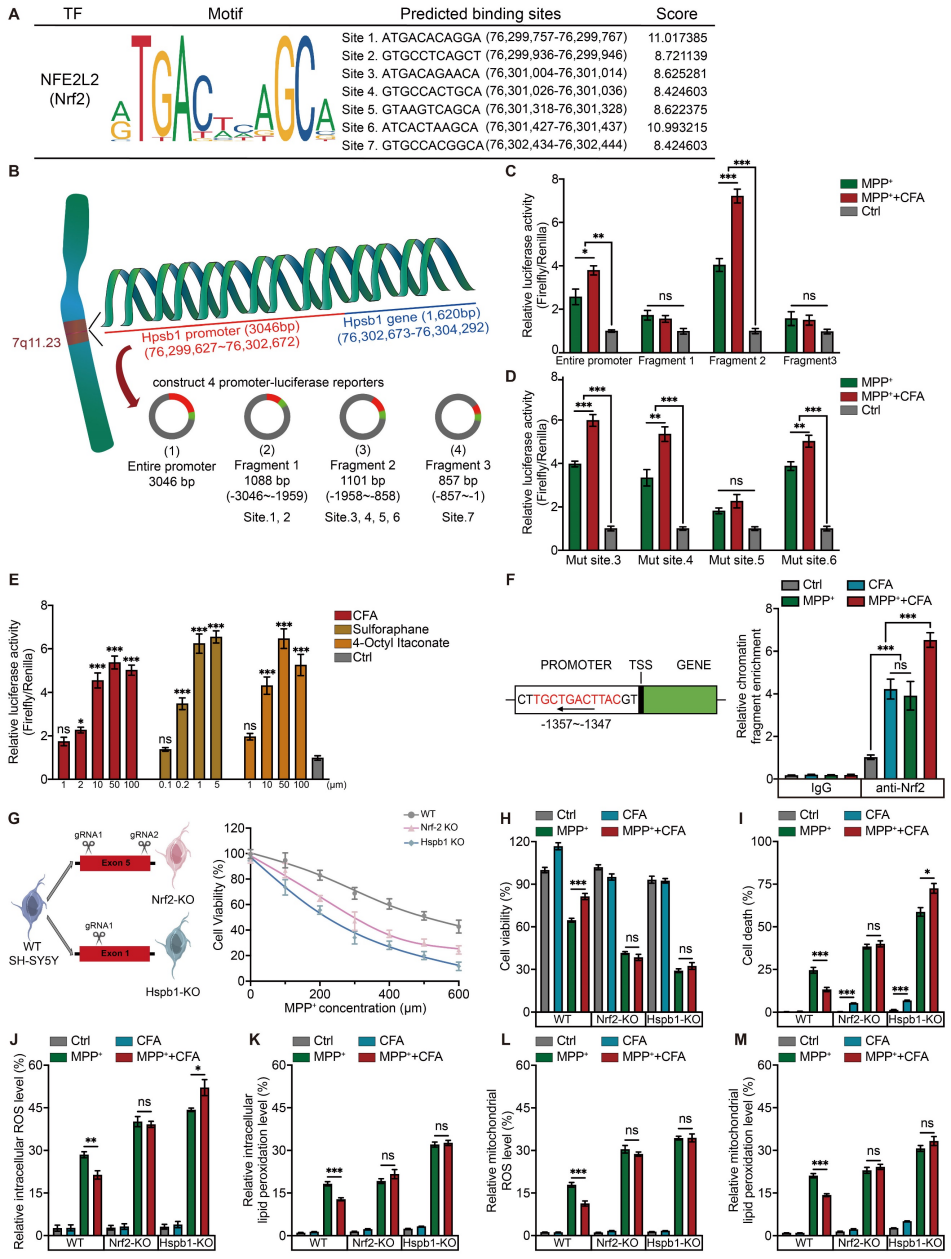
** CFA suppresses ROS and lipid peroxidation accumulation and prevents cell death via the Nrf2-Hspb1 axis. A** Nrf2 binding motif enriched in the promoter regions of genes. The height of the letter is proportional to the frequency of that base. The predicted binding sites in Hspb1's promoter and their probability scores in the promoter of Hspb1. **B** Schematic for the experimental protocol used. The promoter region of the Hspb1 gene (3046 bp) was carried on luciferase reporters. The whole promoter region was divided into 3 fragments and each fragment was carried on luciferase reporters to detect the actual binding sites. Luciferase reporter activity assays were performed in SH-SY5Y cells. The relative luciferase and renilla activity were measured. **C** Relative luciferase and renilla activity of different promoter-reporter were measured and the ratio of luciferase was normalized by renilla. Data are presented as mean ± SEM. Statistics were assessed using one-way ANOVA followed by TUKEY post hoc tests. *p < 0.05, **p < 0.01, ***p < 0.001, ns, not significant (n = 4). **D** Relative luciferase activity was measured after mutation of each potential binding site and normalized by renilla. Data are presented as mean ± SEM. Statistics were assessed using one-way ANOVA followed by TUKEY post hoc tests. *p < 0.05, ***p < 0.001, ns, not significant (n = 5). **E** Relative luciferase activity of fragment 2-reporter was measured after the administration of two other Nrf2 activators besides CFA with a range of concentrations, which are Sulforaphane and 4-Octyl ltaconate. Data are presented as mean ± SEM. Statistics were assessed using one-way ANOVA followed by TUKEY post hoc tests. *p < 0.05, ***p < 0.001, ns, not significant (n = 4). **F** Representative ChIP-qPCR of the enrichment of Nrf2 in the Hspb1 promoter region normalized to IgG in DAergic neurons. Data are presented as mean ± SEM. Statistics were assessed using t test. ***p < 0.001, ns, not significant (n = 4). **G** Gene editing diagram of the Hspb1-KO and Nrf2-KO SH-SY5Y cell lines using CRISPR/Cas9 system. The cytoprotective effects of CFA against different concentrations of MPP^+^ were not observed in Hspb1-KO and Nrf2-KO SH-SY5Y cell lines.** H** Cell viability was quantified in WT and KO cell lines. Data are presented as mean ± SEM. Statistics were assessed using t test. *p < 0.05, **p < 0.01, ***p < 0.001, ns, not significant (n = 8). **I** Cell death ratios were quantified in WT and KO cell lines. Data are presented as mean ± SEM. Statistics were assessed using t test. *p < 0.05, **p < 0.01, ***p < 0.001, ns, not significant (n = 3). **J-M** Quantification of intracellular and mitochondrial ROS, intracellular and mitochondrial lipid peroxidation in WT and KO cell lines. Data are presented as mean ± SEM. Statistics were assessed using t test. *p < 0.05, **p < 0.01, ***p < 0.001, ns, not significant (n = 6).

Previous findings indicated CFA may inhibit ferroptosis by upregulating Hspb1. However, the mechanism by which Nrf2 regulates Hspb1 remains unclear. To verify whether Nrf2 could directly regulate the transcription of Hspb1, we used the JASPAR database to predict Nrf2 binding sites in the Hspb1 promoter region. With a threshold of 80%, we pinpointed 7 potential Nrf2 binding sites within the 3000 bp upstream of the start codon (Figure 5A). Next, we constructed a luciferase reporter with a 3046 bp promoter region and co-transfected this reporter plasmid with Nrf2 overexpressing plasmid into SH-SY5Y cells (Figure 5B).

To precisely identify the active binding sites of Nrf2 on the Hspb1 promoter, the promoter sequence was divided into three fragments, namely Fragment 1 (-3046 to -1959), Fragment 2 (-1958 to -858), Fragment 3 (-857 to -1) and then inserted in luciferase reporter assays. Fragment 2 was found to be the key transcriptional DNA binding region (Figure 5C). Then we mutated the potential DNA binding sites of fragment 2 predicted by JASPAR and transfected them respectively into SH-SY5Y with a Nrf2 overexpressing plasmid. Notably, a mutation at site 5 substantially reduced the CFA-induced luciferase activity (Figure 5D). We also tested other 2 Nrf2 activators, Sulforaphane (SFN), and 4-Octyl Itaconate (4OI), and observed that these activators enhanced the luciferase activity of the fragment 2-reporter in a dose-dependent manner (Figure 5E). These results confirmed that Nrf2 activators robustly promote the interaction of Nrf2 with the Hspb1 promoter. Supporting these findings, chromatin immunoprecipitation coupled with quantitative PCR (ChIP-qPCR) demonstrated that CFA treatment increased Nrf2 binding to the Hspb1 promoter in hPSC-derived DAergic neurons (Figure 5F).

To further elucidate the critical role of the Nrf2/Hspb1 signaling pathway in protecting against cell death, we generated two knockout (KO) SH-SY5Y cell lines: Hspb1-KO and Nrf2-KO, as illustrated in the diagram (Figure 5G). These KO cells, along with WT cells, were treated with 100 µM CFA and exposed to a range of MPP+ concentrations (0 to 600 µM). We observed that the protective effects of CFA were reduced in the absence of Nrf2 and Hspb1 (Figure 5G). We established an optimal concentration of 300 µM MPP+ and 100 µM CFA for subsequent experiments with SY5Y cells. The results of cell viability showed that although Hspb1 and Nrf2 are not critical for cell survival under normal conditions, they are essential for defending against MPP^+^ induced cell death (Figure 5H). Notably, in Hspb1 and Nrf2 KO cells, the cytoprotective effects of CFA were lost, and CFA treatment even tended to enhance cell death, especially in Hspb1-KO cells (Figure 5I). Moreover, the absence of Nrf2 or Hspb1 did not affect intracellular peroxide levels, but it did impair the ability of CFA to mitigate ROS and lipid peroxidation in both the cytoplasm and mitochondria (Figure 5J-5M). Collectively, these results suggest that CFA prevents ferroptosis primarily by inhibiting ROS and lipid peroxidation accumulation, mediated through the Nrf2-dependent upregulation of Hspb1.

### Transient Hspb1 upregulation exerts cytoprotective effects but sustained overexpressing of Hspb1 leads to cell death

**Figure 6:**
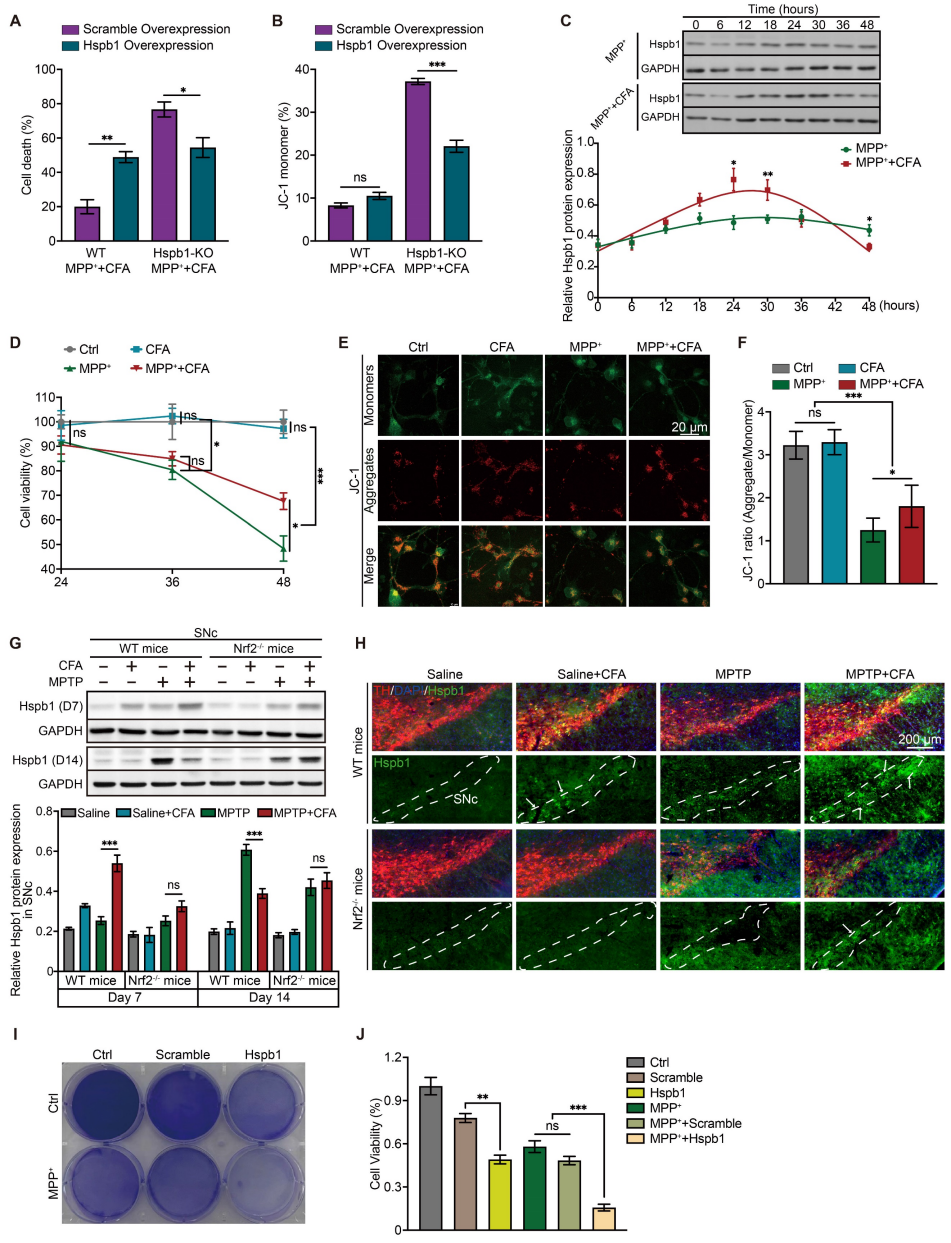
** The function of Hspb1 exhibits dual direction and the durable high expression of Hspb1 may lead to severe cell death. A-B** Quantification of cell death and mitochondrial membrane potential in SH-SY5Y cells pre-transfected with Hspb1 overexpressing or scramble plasmid. Data are presented as mean ± SEM. Statistics were assessed using t test. *p < 0.05, ***p < 0.001, ns, not significant (n = 4). **C** Representative Immunoblotting of the dynamic changes of Hspb1 at different time point lasted for 48 h after treating with CFA during MPP^+^ exposure in WT SH-SY5Y cells. Quantification of Hspb1 was normalized to GAPDH. Data are presented as mean ± SEM. Statistics were assessed using t test. The comparison is between the two groups at each time point. *p < 0.05, **p < 0.01, ***p < 0.001 (n = 4). **D** Cell viability is quantified by MTS assay. Data are presented as mean ± SEM. Statistics were assessed using one-way ANOVA followed by TUKEY post hoc tests. *p < 0.05, ***p < 0.001, ns, not significant (n = 5). **E** Representative images of JC-1 staining in mature midbrain neurons. Red aggregates (525/590 ± 15 nm) represented normal mitochondrial membrane potential. Green monomers (488/530 ± 15 nm) represented depolarized mitochondrial membrane potential. Scale bar, 50 μm. **F** The fluorescence of aggregate/monomer was detected using a fluorescence plate reader and the JC-1 ratio was determined by calculating the ratio of fluorescence intensity by the aggregates to the monomers. Data are presented as mean ± SEM. Statistics were assessed using one-way ANOVA followed by TUKEY post hoc tests. *p < 0.05, ***p < 0.001, ns, not significant (n = 6). **G** Representative Immunoblotting of Hspb1 in SNc lysates from the WT and Nrf2^-/-^ mice on Day 7 and Day 14. Quantification of Hspb1 protein levels in SNc normalized to GAPDH. Data are presented as mean ± SEM. Statistics were assessed using t test. ***p < 0.001, ns, not significant (n = 5). **H** Representative immunofluorescence images of TH (red) and Hspb1 (green) in SNc from different groups on Day 7. Scale bars, 200 μm. **I-J** Representative crystal violet staining image and quantification of cell death rate after incubated in Hspb1-containing medium. Data are presented as mean ± SEM. Statistics were assessed using one-way ANOVA followed by TUKEY post hoc tests. **p < 0.01, ***p < 0.001, ns, not significant (n = 3).

After reintroducing Hspb1 expression in Hspb1-KO cells, we observed a restoration of CFA's therapeutic effectiveness against MPP^+^-induced toxicity, highlighting the critical role of the Hspb1/Nrf2 axis in mediating cell death and mitochondrial dysfunction (Figure 6A-6B). Intriguingly, overexpressing Hspb1 in WT cells led to severe cell death without significantly affecting mitochondrial membrane potential (Figure 6A). This suggested that prolonged Hspb1 expression might trigger cell death through specific pathways. We observed a notable increase in the MPP^+^+CFA group at 24-30 hours, followed by a rapid decline to levels lower than those in the MPP^+^ group within the next 18 hours (Figure 6C). These dynamics indicated that while transient Hspb1 upregulation by CFA might prevent ferroptosis, sustained high expression could promote cell death. Given the characteristic membrane rupture in ferroptosis, we suspected that the subsequent extracellular release of Hspb1 could lead to a form of non-selective cell death. In this study, we also employed an alternative differentiation protocol to generate mature midbrain neurons 102, 103, achieving a composition that closely mimics the human ventral midbrain, comprising approximately 12.10% TH^+^ neurons and 7.88% astrocytes (Figure S4D-S4F). This approach produced fewer dopaminergic neurons compared to the Floor-plate method (86.13%). Significant cell death was not observed at 36 hours; however, extending the incubation period to 48 hours resulted in noticeable cell death and mitochondrial dysfunction. Cell viability and mitochondrial membrane potential dropped to 48.37% and 38.74%, respectively, after exposure to 600μM MPP^+^ (Figure 6D-6F). Collectively, we hypothesized that between 36 and 48 hours, cells produced substances that promote cell death. We speculated that extracellular Hspb1 might serve as one of the significant factors contributing to this process. Notably, while MPP^+^ is typically absorbed by dopaminergic neurons, numerous studies have shown that high concentrations of MPP^+^ can also lead to cell death in non-DAergic neurons, such as primary astrocytes, primary hippocampal neurons, and in cell lines like HT-22 and HMC3 104-107. Under exposure to 600 μM MPP^+^, damage to non-neuronal cells by MPP^+^ is limited, suggesting that cell death in non-DAergic neurons may have other triggers. Extracellular Hspb1 likely serves as one of the significant factors. To further validate these findings, we examined Hspb1 protein levels in the SNc from the MPTP-induced PD model of mice. Immunoblots revealed a significant, Nrf2-dependent increase in Hspb1 by Day 7 in the WT group (Figure 6G). We also observed Hspb1 protein levels in SNc, especially in TH^+^ neurons on Day 7. Consistent with immunoblotting results, after the CFA treatment, Hspb1 was highly expressed in the cytoplasm of DAergic neurons (Figure 6H). We further detected the protein levels of Hspb1 in SNc on Day 14. We also monitored Hspb1 protein levels in the SNc on Day 14, finding that while Hspb1 levels in the MPTP+CFA group had normalized, they continued to increase in the MPTP group (Figure 6G), aligning with prior in vitro findings that CFA's therapeutic effect relies on transient Hspb1 upregulation.

To further explore whether extracellular Hspb1 exacerbates PD by inducing cellular damage, we evaluated its effect on cell death in vitro. SY5Y cells were engineered to overexpress either Hspb1 or a control vector (Scramble), and subjected to multiple freeze-thaw cycles followed by centrifugation to eliminate cellular debris. The supernatant containing Hspb1 or Scramble was then harvested. SY5Y cells were incubated in medium supplemented with this supernatant and 300 μM MPP^+^ for 36 hours. We found that cells treated with the Hspb1-enriched medium displayed significantly decreased cell viability and heightened susceptibility to mitochondrial dysfunction (Figure 6I-6J), indicating that extracellular Hspb1 can directly damage cells.

Although extracellular Hspb1 induces substantial cell death in vitro, the dynamic exchange of interstitial fluid in vivo could prevent sustained high concentrations of extracellular Hspb1, possibly diminishing its role as a primary contributor to cell death in the SNc of PD animal models or patients. Despite this, Hspb1 represents a significant threat to the survival of dopaminergic neurons, particularly those already weakened by mitochondrial dysfunction. This identifies Hspb1 as a crucial determinant of neuronal susceptibility.

### Extracellular Hspb1 as a potential trigger for inflammatory responses

snRNA-seq data and Bulk RNA-seq data indicated that MPTP-induced neural toxicity might also stimulate inflammatory responses (Figure S1D and Figure 3B). Post-mortem analyses of brains from PD patients show accumulation of activated microglia around DAergic neurons, highlighting a strong association between microglial activation and neuronal death in PD 108. The substances released from neuronal membrane ruptures are likely crucial in triggering inflammation, a process notably prominent in pyroptosis, which similarly involves membrane rupture 109.

**Figure 7:**
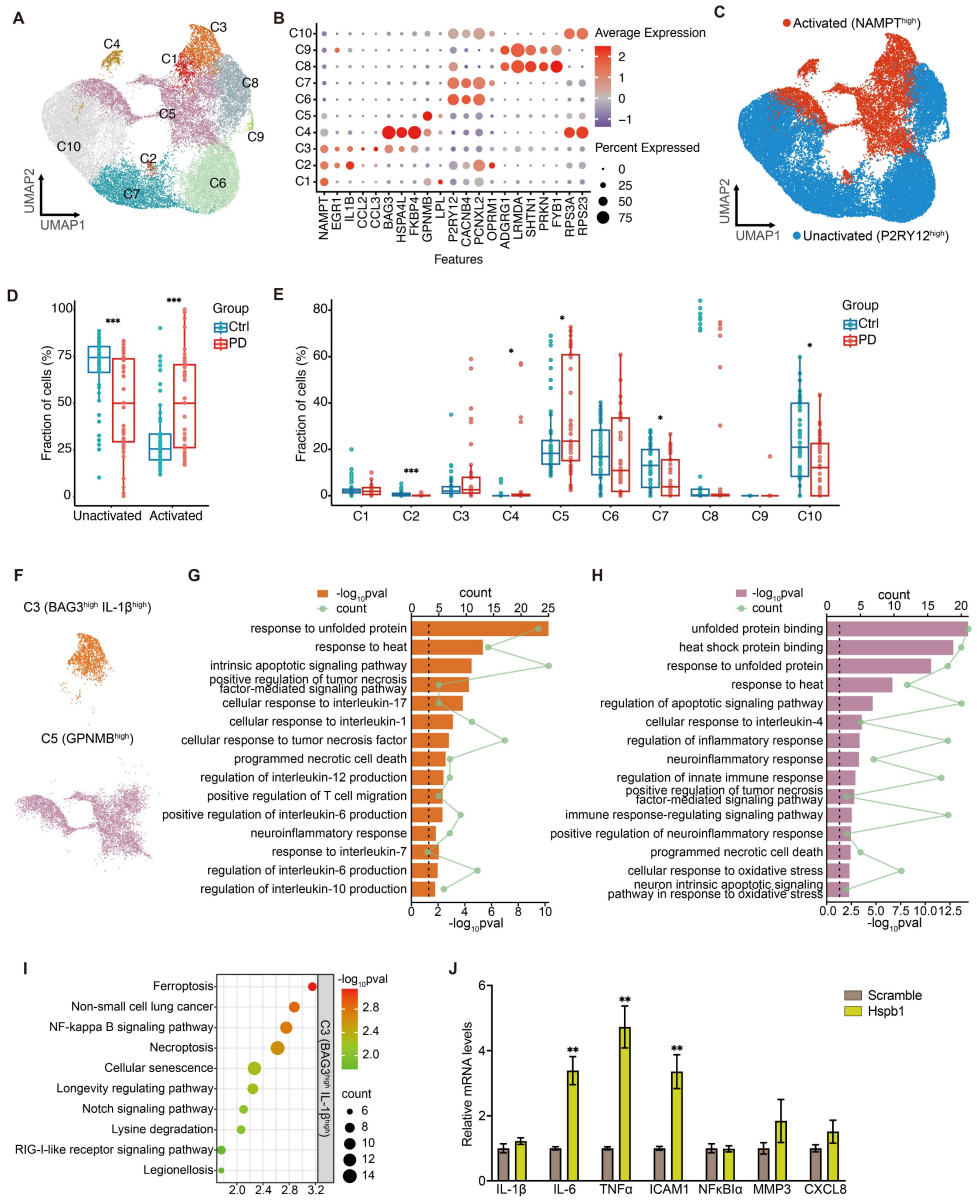
** Extracellular Hspb1 leads to neuroinflammatory response in PD. A** Dot plot visualization of selected biomarker genes identifies 10 distinct microglial subpopulations. **B** UMAP embeddings of microglia, colored by the status of microglia. P2RT12^high^ characterizes the inactivated microglial subpopulation, while NAMPT^high^ characterizes the activated microglial subpopulation. **C** Frequency of inactivated and activated microglia as a proportion of all microglia from PD patients and Ctrl group. Center line indicates the median value, bottom and top hinges represent the 25th and 75th percentiles, respectively and whiskers denote 1.5 × interquartile range. ***p < 0.001; Statistics were assessed using wilcox test. **D** UMAP embeddings of microglia, colored by microglial subpopulations. **E** Frequency of microglial subpopulations as a proportion of all microglia from PD patients and Ctrl group. Center line indicates the median value, bottom and top hinges represent the 25th and 75th percentiles, respectively and whiskers denote 1.5 × interquartile range. *p < 0.05, ***p < 0.001; two-sided t-test. **F-H** GO analysis of DEGs in C3 and C5 microglial subpopulations upregulated in PD. **I** KEGG analysis highlighted several signal pathways regulated upregulated in PD. **J** Relative mRNA expression levels of the pro-inflammatory genes and microglial activation genes in HMC3 cells after incubated in Hspb1-containing medium were measured using qPCR. Data are presented as mean ± SEM. Statistics were assessed using t test. **p < 0.01 (n = 4).

To investigate the role of inflammation in PD, we further analyzed snRNA-seq data and identified ten distinct human microglial subpopulations, using specific microglia-enriched biomarkers (Figure 7A-7B and Figure S7). P2RY12, a marker of microglia, shows significant downregulation in various disease-related inflammatory states, contrasting with the expression of NAMPT, which increases upon immune cell activation 110-113. Using P2RY12 and NAMPT as markers, we categorized microglia into activated (C1-C5) and resting (C6-C10) states (Figure 7C). PD patients exhibited a significant higher proportion of activated (NAMPT^high^) and a lower proportion of resting (P2RY12^high^) microglia compared to the control group (Figure 7D). BAG3, Bcl-2-associated athanogene 3, is upregulated in activated microglia in neurodegenerative diseases 114, 115. It interacts with HSPs, including Hspb1, to mediate the degradation of misfolded proteins 116, playing a role in regulating cell death and cellular stress response 117. IL-1β, a key regulator of neuroinflammation secreted by activated microglia, has been shown to promote the progression of various neurodegenerative diseases and impair learning and memory functions 118.

The C3 microglial subpopulation, characterized by high expression of BAG3 and IL-1β, showed no significant difference in cell numbers between PD patients and controls (Figure 7E-7F). However, the DEGs in C3 microglia from PD patients were significantly enriched for the secretion of and response to the pro-inflammatory factors, including IL-1 and IL-6 (Figure 7G). This suggested that C3 microglial subpopulation may be involved in the neuroinflammatory processes of PD, contributing to the secretion of inflammatory factors and neuroinflammation. Additionally, DEGs in C3 microglia from PD patients were significantly enriched in terms of cell death, suggesting this microglial subpopulation may be prone to cell death in PD. Further KEGG pathway analysis hints that ferroptosis may be a significant mode of cell death present in this subset (Figure 7I). GPNMB, defining the subpopulation C5, is typically upregulated in neurodegenerative diseases and plays a pivotal role in PD, potentially enhancing microglial efficiency in clearing protein aggregates through mechanisms linked to increased autophagy 119, 120. Notably, the proportion of GPNMB^high^ subpopulation (C5) significantly higher in PD patients (Figure 7E). Enrichment analysis of DEGs in C5 subpopulation showed significant enrichment of cell death and inflammatory activation pathways in PD (Figure 7H). Overall, microglia in PD patients exhibit significant signs of activation and proliferation, which could exacerbate the progression of PD through the secretion of pro-inflammatory factors and contribute to surrounding tissue cell death. These microglia may themselves be vulnerable to cell death, with ferroptosis playing a significant role in the C3 subpopulation.

To explore whether extracellular Hspb1 not only promotes neuronal death but also stimulates microglial activation, we treated the human microglial cell line HMC3 with a medium containing Hspb1 for 6 hours. Subsequent analysis revealed significantly increased mRNA levels of pro-inflammatory factors such as IL-6, TNFα, and ICAM1 after exposure to the Hspb1-containing medium (Figure 7J), underscoring extracellular Hspb1's role in microglial activation and inflammatory responses in PD. Additionally, extracellular Hspb1 induced an upregulation of intracellular Hspb1, similar to observations in myocardial ischemia/reperfusion injury 121, 122. Thus, Hspb1 released into the extracellular environment from ruptured neurons can activate microglia in PD, enhance the release of inflammatory factors and potentially exacerbate the progression and severity of PD. Additionally, extracellular Hspb1 poses a risk to the survival of microglial cells, particularly those migrating towards SNc dopaminergic neurons, further complicating the pathological landscape in PD.

## Discussion

Notable findings from 2016 have pinpointed the role of ferroptosis in the MPTP mouse model of PD 123. Further research has established MPTP/MPP^+^ as triggers of neuronal ferroptosis, although the exact mechanisms behind this remain elusive 25, 124-129. Our research has shown that MPTP induces ROS accumulation and lipid peroxidation within mitochondria, as well as the release of Fe^2+^ into the cytoplasm following mitochondrial rupture, thereby exacerbating ferroptosis. Additionally, our study demonstrates that activation of Nrf2 can mitigate MPTP/MPP^+^-induced ferroptosis, primarily by improving mitochondrial function and reducing lipid peroxidation. Importantly, we have identified that the protective effects of Nrf2 activation against ferroptosis are closely linked to the upregulation of Hspb1. Through dual luciferase reporter assays and CHIP-qPCR, we have determined that Nrf2 can directly bind to the promoter region of Hspb1, enhancing its transcriptional activity. Moreover, by employing CRISPR/Cas9 technology to knock out the Hspb1 or Nrf2 genes, we observed that the protective effects conferred by Nrf2 activation were lost. These findings unveiled a novel anti-ferroptosis signaling pathway.

Fascinatingly, CFA treatment caused cytotoxic effects in both Nrf2-KO and Hspb1-KO cells, as indicated by increased rates of cell death, thereby enriching our comprehension of the Nrf2 signaling pathway. Nrf2, an essential antioxidant transcription factor, is maintained at low levels under normal conditions, where it performs non-critical physiological functions. This observation aligns with studies in Nrf2^-/-^ mice, which show no significant differences in lifespan, weight, or behavior compared to their WT counterparts. However, the resistance of Nrf2^-/-^ mice to toxic challenges is markedly lower than that of WT mice, as demonstrated by more severe behavior and lower survival rates in MPTP models. Notably, Nrf2 lacks a specific agonist and is believed to be activated by various cellular stressors, functioning subsequently in the nucleus. Therefore, most small molecular compounds may activate Nrf2 through pathways similar to those activated by MPTP/MPP^+^, without causing irreversible cell damage. Typically, any potential harm from Nrf2 activators is counterbalanced by the cytoprotective effects of Nrf2 activation. Similarly, Hspb1 is minimally expressed under normal physiological conditions, indicating a limited role in essential cellular functions and a negligible direct effect on cell death or proliferation. However, in response to MPTP-induced oxidative stress, Hspb1 expression is substantially increased and regulated by Nrf2, underscoring its role in mitigating oxidative damage. Notably, the in vitro experiments have shown that cellular resistance to oxidative stress is more significantly compromised by Hspb1 deletion than by Nrf2 deletion, underscoring the vital role of Hspb1 in cellular defense against oxidative stress and related damage.

In this study, we unexpectedly discovered that Hspb1 upregulation induced by MPTP did not decrease promptly, and sustained high expression of Hspb1 significantly increases cell death. This observation compels us to reconsider the dual role of Hspb1. Analysis of snRNA-seq data further revealed that Hspb1 is significantly overexpressed across various cell types, a response initially thought to be a cellular self-rescue mechanism. However, our in vitro studies indicated that while overexpression of Hspb1 enhanced resistance in Hspb1-KO cells, it led to increased cell death in WT cells. In experiments with iPSC-derived midbrain cells (comprising 12.1% DAergic neurons), exposure to MPP^+^ caused cell death well beyond the proportion of DAergic neurons, suggesting that prolonged high expression of Hspb1 might inflict secondary damage on neighboring cells in the SNc. Culturing cells in a medium containing Hspb1 resulted in significant cell death and reduced resistance to oxidative stress. Further snRNA-seq analysis showed pronounced neuroinflammation in PD patients and the critical role of extracellular Hspb1 in activating microglia and inducing the secretion of inflammatory factors. These findings delineate a novel pathogenic process in PD where stressed dopaminergic neurons attempt to mitigate stress through Hspb1 overexpression, leading to ferroptosis (membrane rupture) and the release of intracellular Hspb1, which causes secondary cell death in surrounding cells. In this context, Nrf2 agonists can more effectively upregulate Hspb1, clearing intracellular peroxides, alleviating stress, and preventing cell death, thereby mitigating the harmful release of Hspb1. The biphasic role of Hspb1 suggests that its expression levels should be carefully regulated in PD to avoid exacerbating the disease. While Hspb1 overexpression may initially serve as a protective mechanism against cellular stress, sustained high levels can lead to detrimental effects, including increased cell death and neuroinflammation. Thus, therapeutic strategies should focus on precisely regulating Hspb1 expression to harness its protective benefits without triggering harmful side effects.

Overall, this study highlights the crucial role of the Nrf2-Hspb1 pathway in mitigating ferroptosis and safeguarding neurons in PD. Our findings underscore the cytoprotective effects of pharmacologically enhancing Nrf2 transcriptional efficiency, which directly bind to promoter region and upregulate Hspb1 expression, thereby blocking ferroptosis and offering a novel avenue for therapeutic intervention in PD. Additionally, we have discovered the biphasic function of Hspb1, which protects cells during acute stress but may lead to increased cell death when overexpressed over prolonged periods. These findings not only reveal the complexities of cellular responses to oxidative stress but also emphasize the importance of carefully timing Hspb1 regulation in developing treatments for neurodegenerative diseases.

## Supplementary Material

Supplementary figures and tables.
