# Supplementary material for: The biphasic role of Hspb1 on ferroptotic cell death in Parkinson's disease
Source: Theranostics. 2024 Aug 1;14(12):4643–66. doi: 10.7150/thno.98457 (PMC11373631; doi:10.7150/thno.98457)
Supplement: Supplementary file 1 — Supplementary figures and tables. [file thnov14p4643s1.pdf]

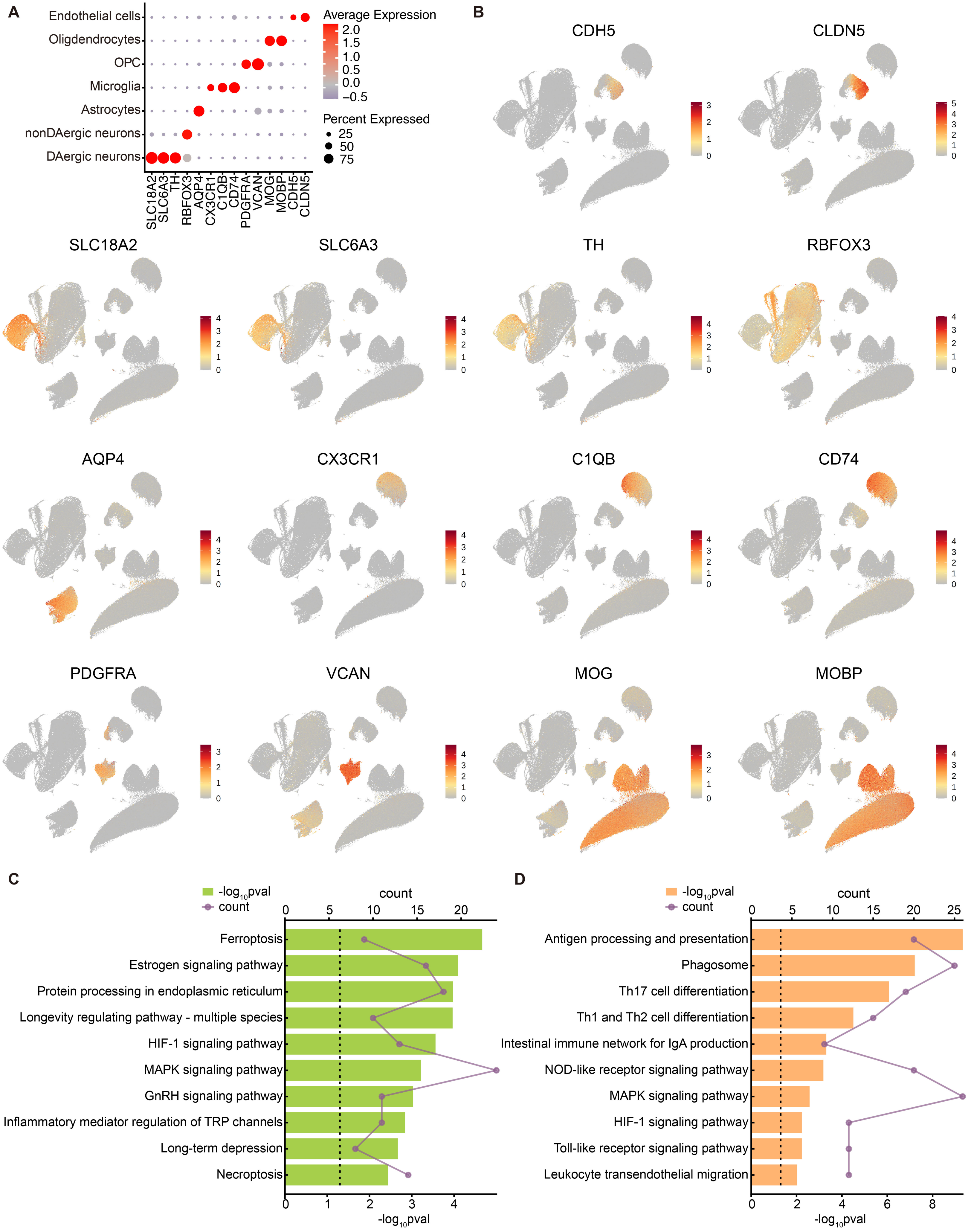

A
Endothelial cells
Oligdendrocytes
OPC
Microglia
Astrocytes
nonDAergic neurons
DAergic neurons
SLC18A2
SLC6A3
TH
RBFOX3
AQP4
CX3CR1
C1QB
CD74
PDGFRA
VCAN
MOG
MOBP
CDH5
CLDN5
Average Expression
Percent Expressed
B
CDH5
CLDN5
SLC18A2
SLC6A3
TH
RBFOX3
AQP4
CX3CR1
C1QB
CD74
PDGFRA
VCAN
MOG
MOBP
C
-log10pval
count
Ferroptosis
Estrogen signaling pathway
Protein processing in endoplasmic reticulum
Longevity regulating pathway - multiple species
HIF-1 signaling pathway
MAPK signaling pathway
GnRH signaling pathway
Inflammatory mediator regulation of TRP channels
Long-term depression
Necroptosis
D
Antigen processing and presentation
Phagosome
Th17 cell differentiation
Th1 and Th2 cell differentiation
Intestinal immune network for IgA production
NOD-like receptor signaling pathway
MAPK signaling pathway
HIF-1 signaling pathway
Toll-like receptor signaling pathway
Leukocyte transendothelial migration

Figure S1 Characterization of different cell types in the midbrain, Related to Figure 1

A Dot plot visualization of the expression of the biomarker genes of different cell types in the different nuclei clusters from midbrain.

B Expression distribution of cell type marker genes on the midbrain cells.

C GO analysis of upregulated DEGs in astrocyte in PD.

D GO analysis of upregulated DEGs in microglia in PD.

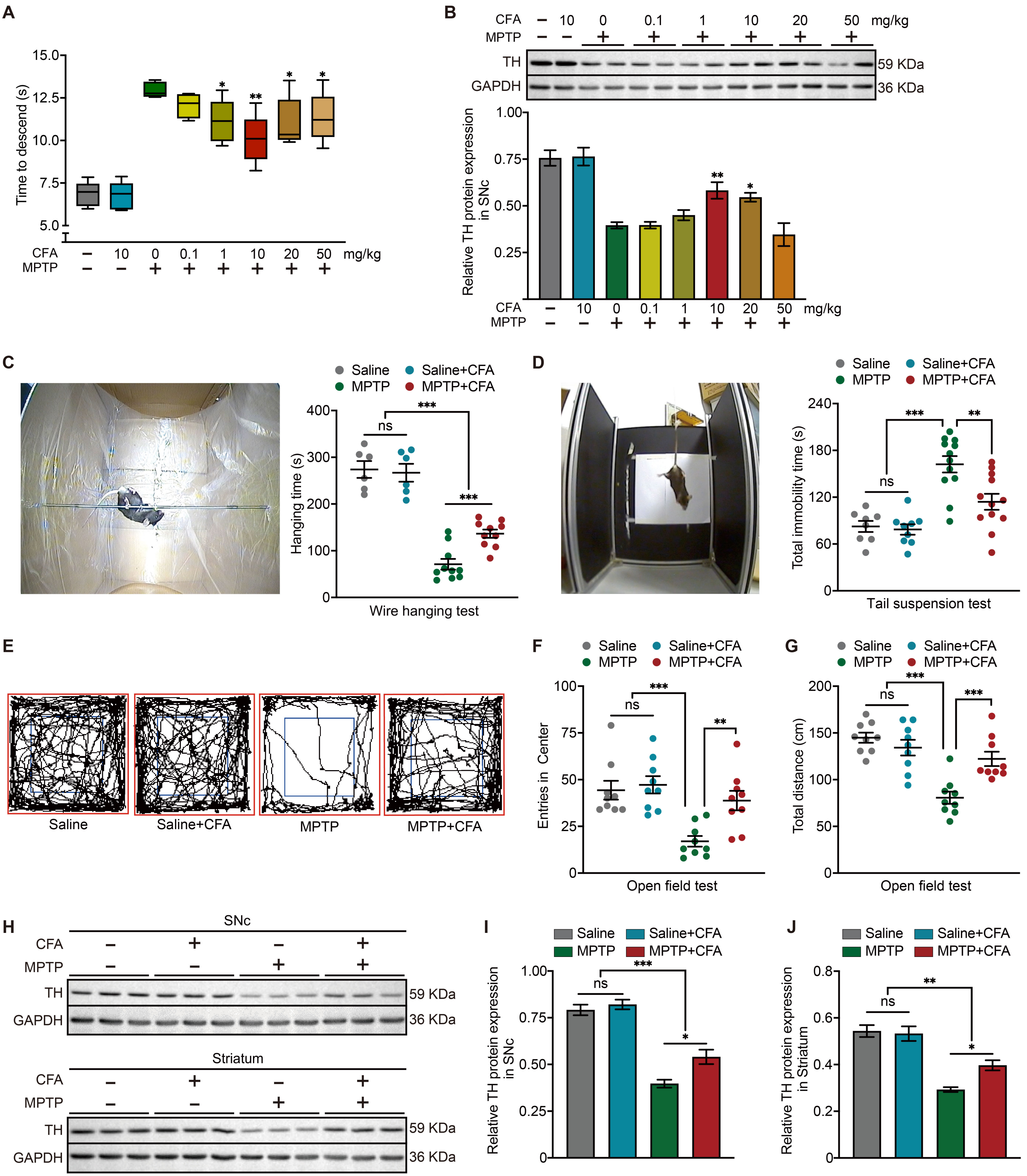

A
Time to descend (s)
15.0
12.5
10.0
7.5
5.0
CFA – 10 0 0.1 1 10 20 50 mg/kg
MPTP – – + + + + + +
B
CFA – 10 0 0.1 1 10 20 50 mg/kg
MPTP – – + + + + + +
TH
GAPDH
59 KDa
36 KDa
Relative TH protein expression in SNc
1.00
0.75
0.50
0.25
CFA – 10 0 0.1 1 10 20 50 mg/kg
MPTP – – + + + + + +
C
Saline
Saline+CFA
MPTP
MPTP+CFA
Hanging time (s)
400
300
200
100
0
ns
***
Wire hanging test
D
Saline
Saline+CFA
MPTP
MPTP+CFA
Total immobility time (s)
240
180
120
60
0
ns
***
**
Tail suspension test
E
Saline
Saline+CFA
MPTP
MPTP+CFA
F
Saline
Saline+CFA
MPTP
MPTP+CFA
Entries in Center
100
75
50
25
0
ns
***
**
Open field test
G
Saline
Saline+CFA
MPTP
MPTP+CFA
Total distance (cm)
200
150
100
50
0
ns
***
Open field test
H
SNc
CFA – + – +
MPTP – – + +
TH
GAPDH
59 KDa
36 KDa
Striatum
CFA – + – +
MPTP – – + +
TH
GAPDH
59 KDa
36 KDa
I
Saline
Saline+CFA
MPTP
MPTP+CFA
Relative TH protein expression in SNc
1.00
0.75
0.50
0.25
0
ns
***
*
J
Saline
Saline+CFA
MPTP
MPTP+CFA
Relative TH protein expression in Striatum
0.8
0.6
0.4
0.2
0
ns
**
*

Figure S2: Preliminary exploration of effect and the best concentration of CFA for in vivo and in vitro study, Related to Figure 2

A Pole test was performed to identify the ideal concentration of CFA for abrogating motor deficits. We set a concentration gradient from 0.1 mg/kg to 50 mg/kg. Data are presented as mean ± SEM. Statistics were assessed using t test (compared with MPTP group). *p < 0.05, **p < 0.01 (n =5).

B Representative immunoblots and quantification of TH normalized to GAPDH in SNc (-3.64nm from the bregma) with the dose of CFA from 0.1 to 50 mg/kg. Data are presented as mean ± SEM. Statistics were assessed using t test (compared with MPTP group). *p < 0.05, **p < 0.01 (n =5).

C-D The final concentration was determined to be 10 mg/kg. To further confirm the neuroprotective effect of CFA, the motor function of mice was assessed using wire hanging test and tail suspension test. Data are presented as mean ± SEM. Statistics were assessed using t test. *p < 0.05, **p < 0.01, ***p < 0.001, ns, not significant (n = 6–12 mice per group).

E-G The Open field test was conducted to evaluate MPTP-induced depressive-like symptoms and the therapeutic effects of CFA. Images were included to depict the representative activity traces in the open field during the 10-minute test period. The red square represented the entire area, and the blue represented the central area. Entries in center and total distance were expressed as mean ± SEM. Statistics were assessed using one-way ANOVA followed by TUKEY post hoc tests. **p < 0.01, ***p < 0.001, ns, not significant (n = 9).

H-J Representative Immunoblotting of TH from brain lysates of the mice in different groups. SNc and striatum lysates were harvested after behavior tests. Quantification of TH protein levels from SNc and striatum lysates of the mice were normalized to GAPDH. Data are presented as mean ± SEM. Statistics were assessed using one-way ANOVA followed by Tukey post hoc tests. **p < 0.01, ***p < 0.001, ns, not significant (n = 8)

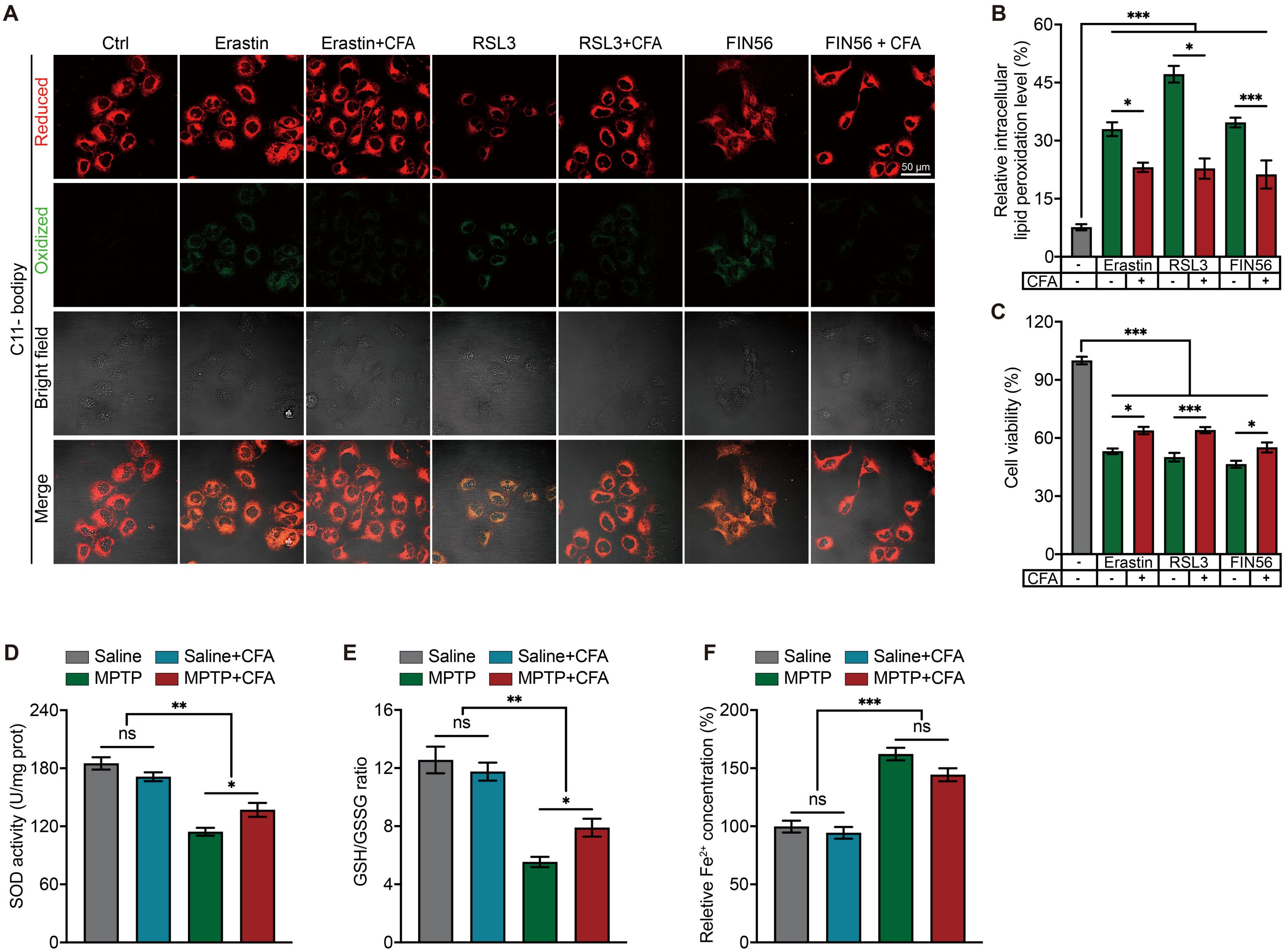
A
Ctrl
Erastin
Erastin+CFA
RSL3
RSL3+CFA
FIN56
FIN56 + CFA
C11- bodipy
Reduced
Oxidized
Bright field
Merge
50 μm
B
Relative intracellular lipid peroxidation level (%)
Erastin
RSL3
FIN56
CFA
C
Cell viability (%)
D
Saline
Saline+CFA
MPTP
MPTP+CFA
SOD activity (U/mg prot)
ns
E
GSH/GSSG ratio
F
Reletive Fe^{2+} concentration (%)

Figure S3: CFA blocks ferroptosis induced by canonical ferroptosis activators, Related to Figure 3

A Representative immunofluorescence images of C11-BODIPY staining. Scale bars, 20 μm. Lipid peroxidation was induced by three ferroptosis inducers (Erastin 10 μM, RSL3 10 μM, FIN56 1 μM) for 24h. Erastin is the most popular ferroptosis inducer, that directly inhibits cystine/glutamate antiporter system Xc- activity and decreases the import of cystine, leading to glutathione (GSH) depletion. RSL3 and FIN56 decrease GPX4 protein levels, leading to the induction of ferroptosis. RSL3 directly inhibits GPX4, causing a decrease in protein levels of GPX4 and an accumulation of lipid peroxidation. FIN56 targets GPX4, causing GPX4 protein degradation and suppression of the lipophilic antioxidant CoQ10 generation. Both RSL3 and FIN56 would ultimately cause ferroptosis.

B Quantification of intracellular lipid peroxidation. Data are presented as mean ± SEM. Statistics were assessed using one-way ANOVA followed by TUKEY post hoc tests. *p < 0.05, ***p < 0.001, ns, not significant (n = 4).

C Cell viability is quantified by MTS assay. Data are presented as mean ± SEM. Statistics were assessed using one-way ANOVA followed by TUKEY post hoc tests. *p < 0.05, ***p < 0.001, ns, not significant (n = 4).

D-F Quantification of SOD activity, GSH/GSSG ratio, and Fe^{2+} levels in SNc lysates in the different groups in mouse PD model. Data are presented as mean ± SEM. Statistics were assessed using one-way ANOVA followed by TUKEY post hoc tests. *p < 0.05, **p < 0.01, ***p < 0.001, ns, not significant (n = 5 for SOD activity; n= 4 for GSH/GSSG ratio; n= 8 for Fe^{2+} levels).

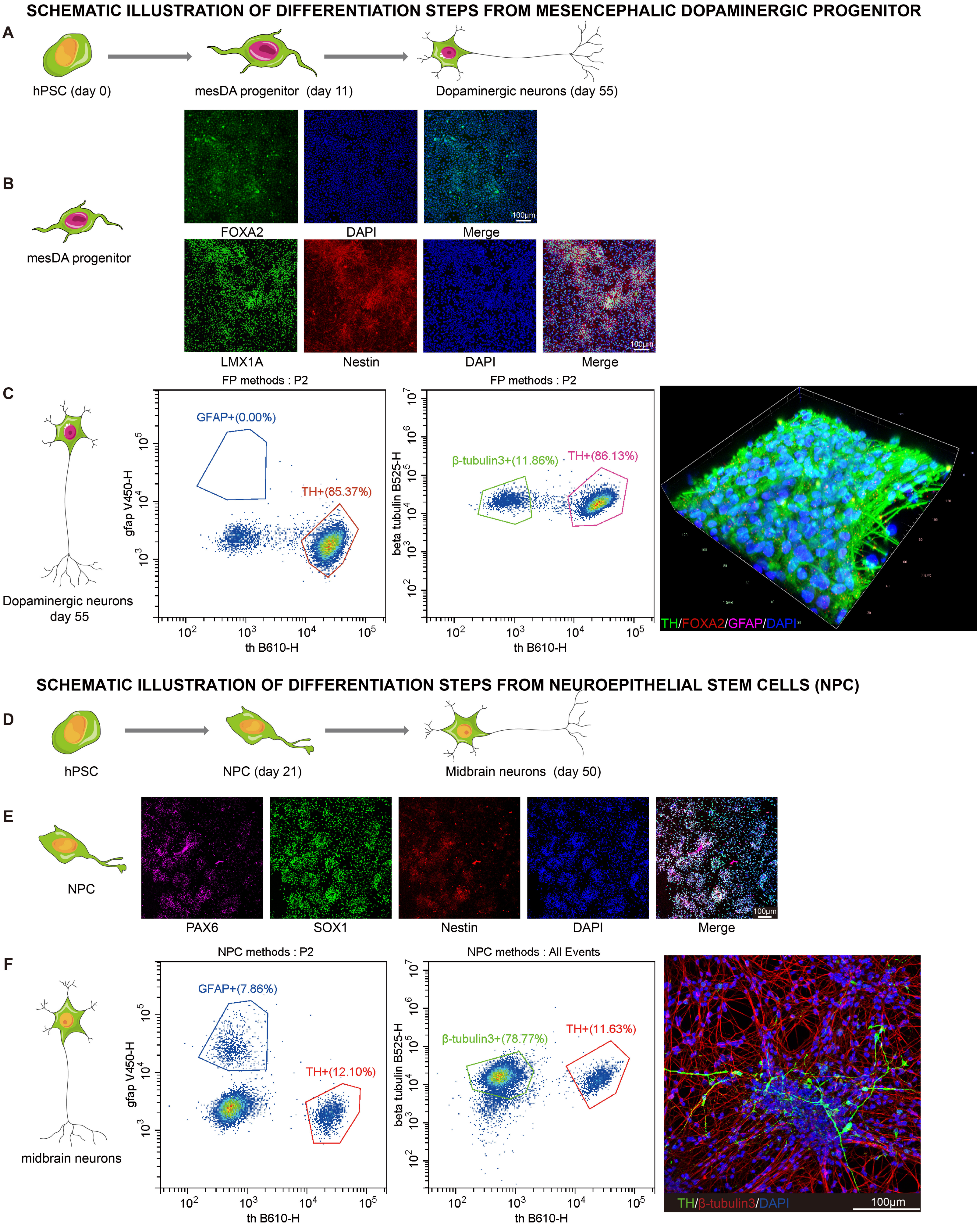

SCHEMATIC ILLUSTRATION OF DIFFERENTIATION STEPS FROM MESENCEPHALIC DOPAMINERGIC PROGENITOR
A
hPSC (day 0)
mesDA progenitor (day 11)
Dopaminergic neurons (day 55)
B
mesDA progenitor
FOXA2
DAPI
Merge
100μm
LMX1A
Nestin
DAPI
Merge
100μm
C
Dopaminergic neurons
day 55
FP methods : P2
GFAP+(0.00%)
TH+(85.37%)
gfap V450-H
th B610-H
FP methods : P2
β-tubulin3+(11.86%)
TH+(86.13%)
beta tubulin B525-H
th B610-H
TH/FOXA2/GFAP/DAPI
SCHEMATIC ILLUSTRATION OF DIFFERENTIATION STEPS FROM NEUROEPITHELIAL STEM CELLS (NPC)
D
hPSC
NPC (day 21)
Midbrain neurons (day 50)
E
NPC
PAX6
SOX1
Nestin
DAPI
Merge
100μm
F
midbrain neurons
NPC methods : P2
GFAP+(7.86%)
TH+(12.10%)
gfap V450-H
th B610-H
NPC methods : All Events
β-tubulin3+(78.77%)
TH+(11.63%)
beta tubulin B525-H
th B610-H
TH/β-tubulin3/DAPI
100μm

Figure S4: Two differentiation strategies of dopaminergic neurons used in this study, Related to Figure 4

A Diagram of the differentiation process of DAergic neurons.

B Representative immunofluorescence images of FOXA2$^+$, LMX1A$^+$, and Nestin$^+$ DAergic progenitor cells at Day 11 of differentiation.

C Representative flow cytometry images exhibited the proportion of GFAP$^+$ astrocytes, β-tubulinⅢ$^+$/TH- neurons and TH$^+$ DAergic neurons in this differentiation strategy.

D Representative immunofluorescence 3D images of hPSC-derived DAergic neurons, depicting TH (green), FOXA2 (red), GFAP (magenta) and DAPI (blue). At Day 55, hPSC-derived DAergic neurons expressed TH in the cytoplasm and expressed low levels of FOXA2 in the nucleus. There was no astrocyte (GFAP positive) observed using this differentiation method.

E Diagram of the differentiation process of NPC-derived dopaminergic neurons.

F Representative immunofluorescence images of PAX6$^+$, SOX1$^+$, and Nestin$^+$ neural progenitor cells at Day 21 of differentiation.

G Representative flow cytometry images exhibited the proportion of GFAP$^+$ astrocytes, β-tubulinⅢ$^+$ neurons and TH$^+$ dopaminergic neurons at Day 50 in this differentiation strategy.

H Representative immunofluorescence images of mature midbrain neurons. Scale bars, 100 μm.

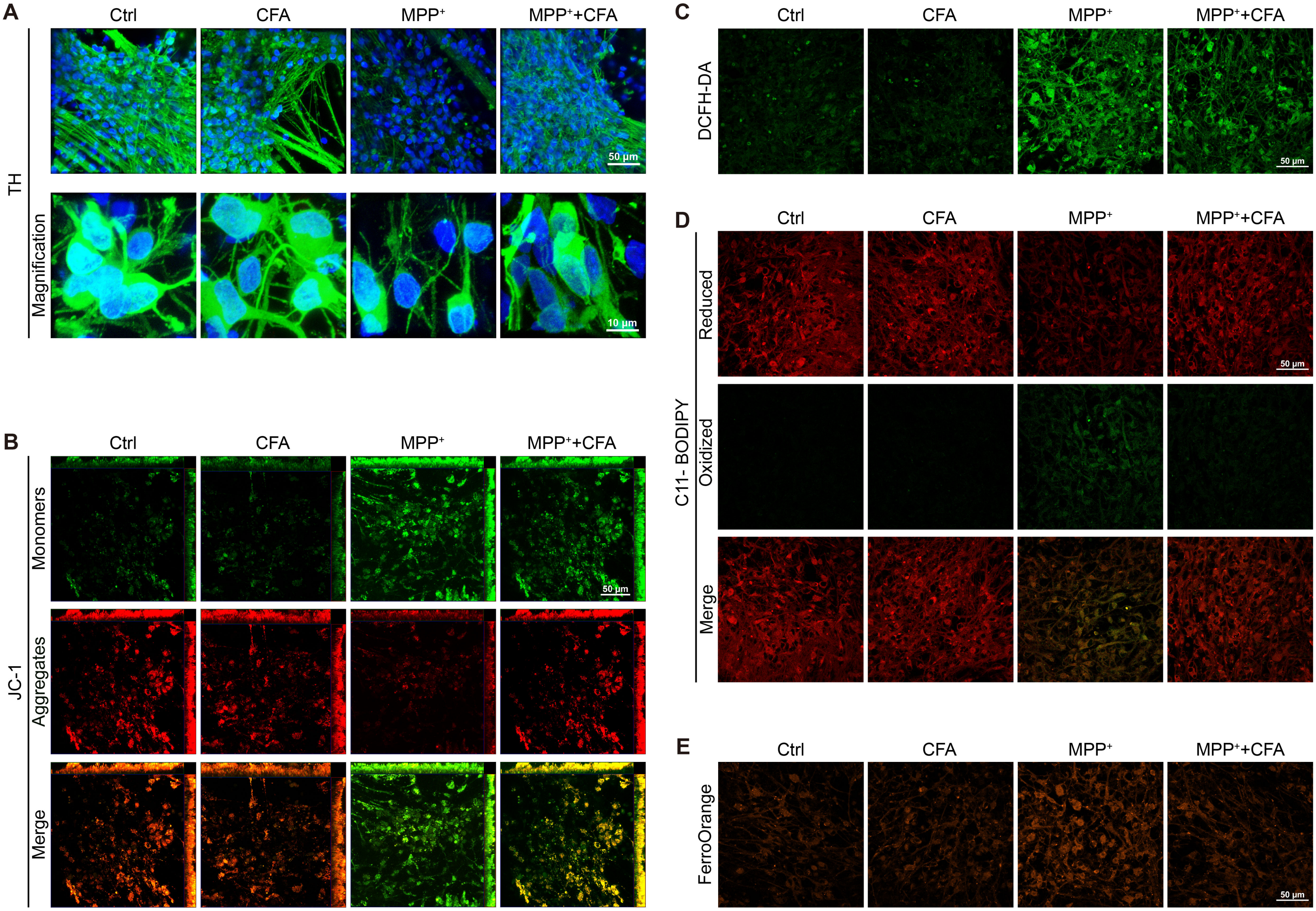

A
Ctrl
CFA
MPP+
MPP+ +CFA
TH
Magnification
50 μm
10 μm
B
Ctrl
CFA
MPP+
MPP+ +CFA
JC-1
Monomers
Aggregates
Merge
50 μm
C
Ctrl
CFA
MPP+
MPP+ +CFA
DCFH-DA
50 μm
D
Ctrl
CFA
MPP+
MPP+ +CFA
C11- BODIPY
Reduced
Oxidized
Merge
50 μm
E
Ctrl
CFA
MPP+
MPP+ +CFA
FerroOrange
50 μm

Figure S5: The cytoprotective effect of Nrf2 activation against MPP^+-induced ferroptosis in DAergic neurons, Related to Figure 4

A Representative immunofluorescence 3D images of neurons, depicting TH^+ (green) and DAPI (blue) of different groups. Scale bars are as indicated in low (50μm) and high magnification (100μm) images, respectively.

B Representative images of JC-1 staining in DAergic neurons. Red aggregates represented normal mitochondrial membrane potential. Green monomers represented depolarized mitochondrial membrane potential. Scale bar, 50μm.

C Representative images of intracellular ROS levels using DCFH-DA staining (488/535 ± 30 nm). Scale bar, 50μm.

D Representative images of intracellular lipid peroxidation levels using C11-BODIPY staining, which showed the reduced C11-BODIPY (565/610 ± 30 nm) and the oxidized C11-BODIPY (488/535 ± 30 nm).

E Representative images of intracellular Fe^{2+} levels using FerrOrange staining (543/580 ± 20 nm). Scale bar, 50μm.

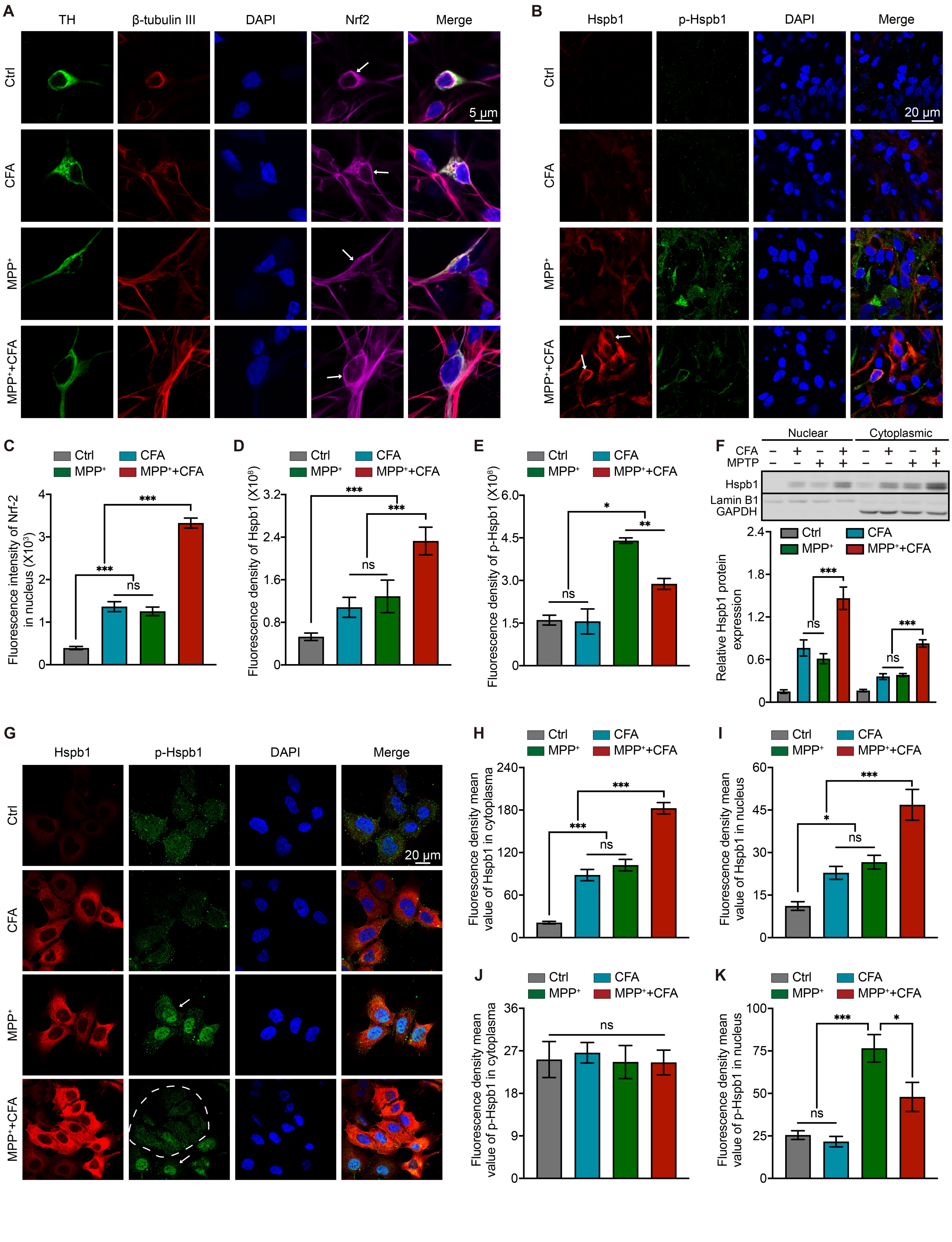
A
TH
β-tubulin III
DAPI
Nrf2
Merge
Ctrl
CFA
MPP+
MPP++CFA
5 μm
B
Hspb1
p-Hspb1
DAPI
Merge
20 μm
C
Ctrl CFA MPP+ MPP++CFA
Fluorescence intensity of Nrf-2 in nucleus (X10³)
D
Fluorescence density of Hspb1 (X10⁸)
E
Fluorescence density of p-Hspb1 (X10⁸)
F
Nuclear
Cytoplasmic
CFA
MPTP
Hspb1
Lamin B1
GAPDH
Relative Hspb1 protein expression
G
Hspb1
p-Hspb1
DAPI
Merge
20 μm
H
Fluorescence density mean value of Hspb1 in cytoplasma
I
Fluorescence density mean value of Hspb1 in nucleus
J
Fluorescence density mean value of p-Hspb1 in cytoplasma
K
Fluorescence density mean value of p-Hspb1 in nucleus
ns
*
**
***

Figure S6: Pharmacological augment of Nrf2 nuclear translocation promotes Hspb1 expression in DAergic neurons and SH-SY5Y cells, Related to Figure 4

A Representative immunofluorescence images of TH, β-tubulin III, DAPI, and Nrf2 staining in NPC-derived midbrain DAergic neurons. Arrows indicated the nuclear translocation efficiency of Nrf2 in different groups. Scale bars, 5μm.

B Representative immunofluorescence images of Hspb1, p-Hspb1, and DAPI staining in DAergic progenitor derived midbrain DAergic neurons. Arrows indicated the upregulated Hspb1 in nucleus of the neurons in MPP^+ + CFA group. Scale bars, 20μm.

C Quantification of Nrf2 protein in the nucleus by measuring the fluorescent density of Nrf2 co-localized with DAPI. Data are presented as mean ± SEM. Statistics were assessed using one-way ANOVA followed by TUKEY post hoc tests. ***$p < 0.001$, ns, not significant ($n = 5$).

D-E Quantification of intracellular Hspb1 and p-Hspb1 protein levels by measuring corresponding fluorescent density. Data are presented as mean ± SEM. Statistics were assessed using one-way ANOVA followed by TUKEY post hoc tests. *$p < 0.05$, **$p < 0.01$, ***$p < 0.001$, ns, not significant ($n = 4$).

F Representative Immunoblotting of Hspb1 in fractionated nucleus and cytoplasm in DAergic progenitor derived DAergic neurons. Quantification of Hspb1 protein levels in cytoplasm was normalized to GAPDH. Nuclear fractions were verified and normalized by Lamin B1. Data are presented as mean ± SEM. Statistics were assessed t tests. ***$p < 0.001$, ns, not significant ($n = 4$).

G Representative immunofluorescence images of Hspb1, p-Hspb1 and DAPI in SH-SY5Y cells. Scale bars, 20μm.

H-I Quantification of intracellular Hspb1 protein levels in the nucleus and cytoplasm by fluorescent density. Data are presented as mean ± SEM. Statistics were assessed using one-way ANOVA followed by TUKEY post hoc tests. *$p < 0.05$, ***$p < 0.001$, ns, not significant ($n = 8$).

J-K Quantification of intracellular p-Hspb1 protein levels in the nucleus and cytoplasm by fluorescent density. Data are presented as mean ± SEM. Statistics were assessed using one-way ANOVA followed by TUKEY post hoc tests. *$p < 0.05$, ***$p < 0.001$, ns, not significant ($n = 7$).

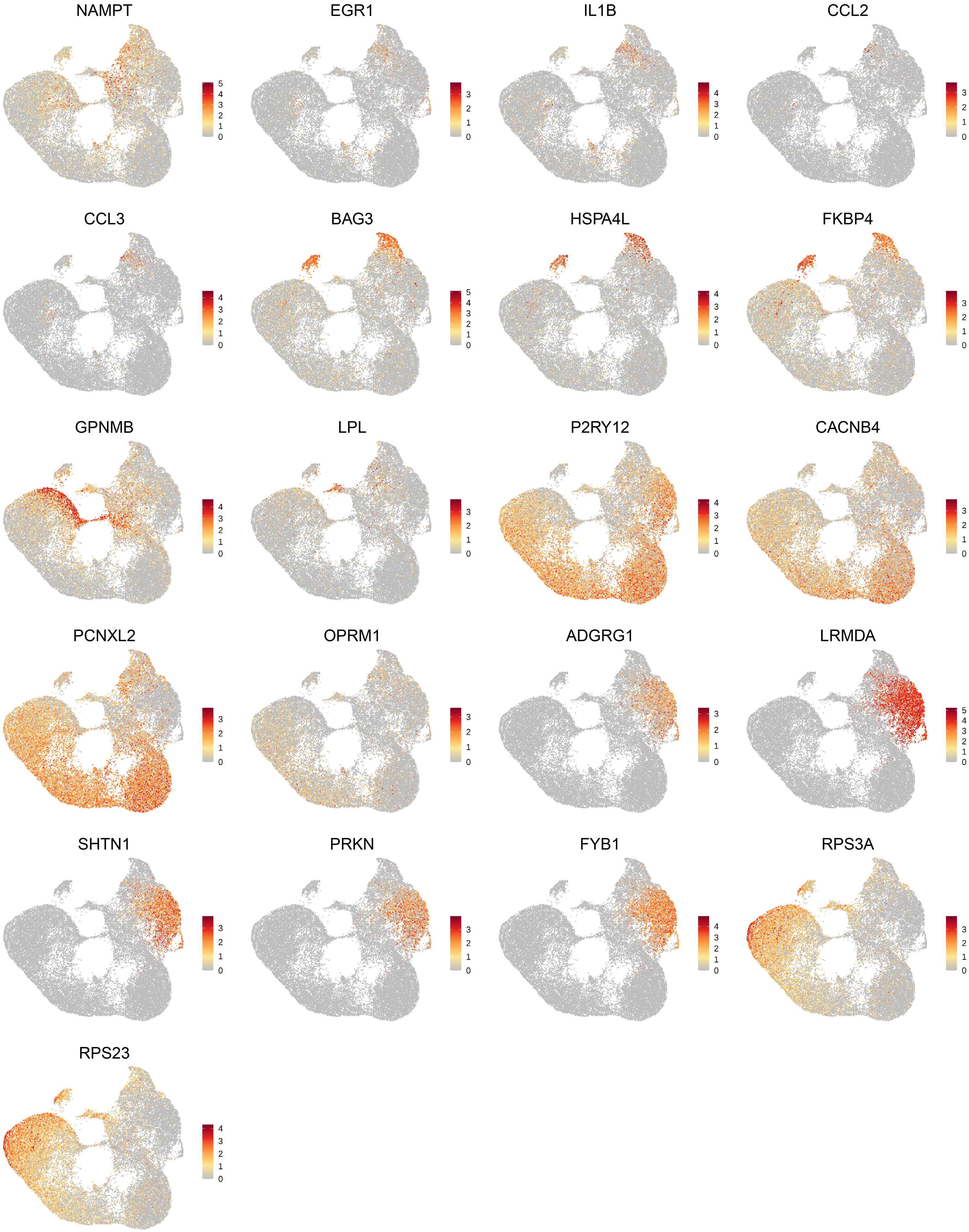
NAMPT
EGR1
IL1B
CCL2
CCL3
BAG3
HSPA4L
FKBP4
GPNMB
LPL
P2RY12
CACNB4
PCNXL2
OPRM1
ADGRG1
LRMDA
SHTN1
PRKN
FYB1
RPS3A
RPS23

Figure S7: Characterization of microglial subpopulation in the midbrain, Related to Figure 7

Expression distribution of marker genes of microglial subpopulations on the midbrain cells.

Main tables and corresponding titles and legends

Table S1 Reagents and proteins

Table S2 Antibodies

Table S3 Primers for PCR

**REAGENT or RESOURCE**	**SOURCE**	**IDENTIFIER**
MPTP hydrochloride	Sigma	M0896-100MG
MPP^+ iodide	Sigma	D048-100MG
Erastin	APEXBIO	B1524
FIN56	MedChemExpress	HY-103087
(1S,3R)-RSL3	MedChemExpress	HY-100218A
Coniferaldehyde	MedChemExpress	HY-N2535
Sulforaphane	MedChemExpress	HY-13755
4-Octyl Itaconate	MedChemExpress	HY-112675
0.5M EDTA in DPBS	Nuwacell	RP01007
Cryopreservation Medium	Nuwacell	RP01003
ncTarget hPSC Medium	Nuwacell	RP01020
mTeSR™ Plus	Stemcell	100-0276
ROCKi	Nuwacell	RP01008
Accutase	Stemcell	7920
Poly-L-Ornithine	Sigma	P4957
Matrigel	Corning	354277
Laminin-521	BioLamina	LN521
Laminin-111	Sigma	L2020
GlutaMAX	Thermo Fisher Scientific	35050061
Neurobasal	Thermo Fisher Scientific	21103049
DMEM/F-12	Thermo Fisher Scientific	11320033
N-2 supplement	Thermo Fisher Scientific	17502048
B-27 supplement W/O vit.A	Thermo Fisher Scientific	12587010
Noggin	R&D	3344-NG-050
SHH C24II	MedChemExpress	HY-P7407
SB 431542	APExBIO	A8249
CHIR99021	Miltenyi	130-106-539
Purmorphamine	Miltenyi	130-104-465
DAPT	R&D	2634
TGFβ3	R&D	243-B3
GDNF	R&D	212-GD-050
BDNF	Abcam	ab206642
Ascorbic acid	Sigma	A4403-100MG
dibutyryl cAMP (cAMP)	Aladdin	D124575
STEMdiff™ SMADi Neural Induction Kit	Stemcell	08582
STEMdiff™ Midbrain Neuron Differentiation Kit	Stemcell	100-0038
STEMdiff™ Midbrain Neuron Differentiation Kit	Stemcell	100-0041
Live Cell Imaging Solution	Thermo Fisher Scientific	A14291DJ
MEM EARLES 500ML	Thermo Fisher Scientific	C11095500BT
F12	Thermo Fisher Scientific	C11765500BT
Sodium Pyruvate	Thermo Fisher Scientific	11360070
MEM NEAA	Thermo Fisher Scientific	11140050
Penicillin-Streptomycin (10,000 U/mL)	Thermo Fisher Scientific	15140122
FBS	Thermo Fisher Scientific	10091148
Trypsin-EDTA (0.25%)	Thermo Fisher Scientific	C25200072
Lipofectamine 2000	Thermo Fisher Scientific	11668019
Mycoplasma Detection Kit	Yeasen	40612ES25

BCA protein assay kit	Thermo Fisher Scientific	23225
JC-1 assay Kit	Abcam	ab113850
Mitochondrial Complex I Activity Colorimetric Assay Kit	Abcam	ab287847
MTS assay kit	Promega	G5421
Ferrous iron assay kit	Elabscience	E-BC-K773-M
MDA assay kit	Sloarbio	BC0025
Total SOD assay kit	Beyotime	S0101S
GSH and GSSG Assay Kit	Beyotime	S0053
Propidium Iodide	Yeasen	40711ES10
Calcein	Beyotime	C2012
DCFH-DA	MedChemExpress	HY-D0940
MitoSOX	ABclonal	RM02822
BODIPY581/591 C11	ABclonal	RM02821
MitoPeDPP	DOJINDO	M466
FerroOrange	DOJINDO	F374
Dual Luciferase Reporter Assay Kit	Vazyme	DL101-01
Sonication ChIP Kit	ABclonal	RK20258
Protein A/G magnetic beads	ABclonal	RM09008
Total RNA Extraction Kit	Transgen	FE201
Strand cDNA Synthesis Kit	Vazyme	R312
Taq Pro Universal SYBR qPCR Master Mix	Vazyme	Q712
Nuclear and Cytoplasmic Protein Extraction Kit	Transgen	DE201
Mitochondrial isolation kit	Proteintech	PK10016

Antibodies		SOURCE	IDENTIFIER
Rabbit anti-TH	1:1000 for Immunoblotting	Milipore	ab152
	1:1500 for IHC		
	1:100 for ICC		
Mouse Alexa Fluor 594 anti-TH	1:1000 for IHC	Biolegend	818002
	1:100 for ICC and Flow Cytometry		
Rabbit anti-Slc7a11	1:1000 for Immunoblotting	Abclonal	A2413
Rabbit anti-Gpx4	1:2000 for Immunoblotting	Abclonal	A11243
Rabbit anti-DAT	1:500 for Immunoblotting	Abclonal	A15236
Mouse anti-GAPDH	1:4000 for Immunoblotting	Applygen	C1212-1
Rabbit anti-SOX1	1:200 for ICC	Abcam	ab109290
Mouse anti-PAX6	1:100 for ICC	Abcam	ab78545
Mouse anti-EN1	1:200 for ICC	DSHB	AB_528219
Rabbit anti-LMX1A	1:1000 for ICC	Abcam	ab139726
Mouse Alexa Fluor 594 anti-Nestin	1:100 for ICC	Biolegend	656804
Mouse Brilliant Violet 421 anti-GFAP	1:20 for ICC and Flow Cytometry	Biolegend	644710
Mouse Alexa Fluor 488 anti-beta III Tubulin	1:200 for ICC and Flow Cytometry	Biolegend	657403
Rabbit anti-FOXA2	1:50 for ICC	Abcam	ab60721
Mouse anti-Hspb1	1:20000 for Immunoblotting	Proteintech	66767-1-Ig
	1:50 for ICC		
Rabbit anti-Hspb1	1:5000 for Immunoblotting	Proteintech	18284-1-AP
	1:1000 for IHC		
Rabbit anti-Hspb1 (phospho S78)	1:150 for ICC	abcam	ab32501
Rabbit anti-Nrf2	1:50 for CHIP	Abcam	ab62352
Rabbit Alexa Fluor594 anti-Nrf2	1:100 for ICC	Abcam	ab206890
Mouse anti-Lamin B1	1:1000 for Immunoblotting	Proteintech	66095-1-Ig
Rabbit anti-COXIV	1:2000 for Immunoblotting	Proteintech	11242-1-AP
Goat Alexa Fluor 488 anti-Rabbit	1:1000 for IHC	Abcam	ab150081
	1:100 for ICC		
Goat Alexa Fluor 647 anti-Mouse	1:1000 for IHC	Abcam	ab150119
	1:100 for ICC		
Rabbit Anti-Control IgG	1:50 for CHIP	Abclonal	AC005

Primer for PCR	Forward Primer 5'- 3'	Reverse Primer 5'- 3'
Nrf2 (Mouse)	TTCTTTCAGCAGCATCCTCTCCAC	ACAGCCTTCAATAGTCCCGTCCAG
Srebf1 (Mouse)	TGACCCGGCTATTCCGTGA	CTGGGCTGAGCAATACAGTTC
Hsf1 (Mouse)	CGAGTGGGAACAGCTTCCA	ACTTGGGCAGCACCTCCTT
H-ferritin (Mouse)	GCTGAATGCAATGGAGTGTGCA	GGCACCCATCTTGCGTAAGTTG
Sod2 (Mouse)	CAGACCTGCCTTACGACTATGG	CTCGGTGGCGTTGAGATTGTT
Hspb1 (Mouse)	CACTGGCAAGCACGAAGAAAG	GCGTGTATTTCCGGGTGAAG
Hspa5 (Mouse)	GAA ATGGCCCAGTGAGAAAA	CTTCCACGTTGCTGACTTGA
Slc7a11 (Mouse)	CTATTTTACCACCATCAGTGCG	ATCGGGACTGCTAATGAGAATT
Gpx4 (Mouse)	GAGGCAAGACCGAAGTAAACTAC	CCGAACTGGTTACACGGGAA
GAPDH (Mouse)	AGGTCGGTGTGAACGGATTTG	TGTAGACCATGTAGTTGAGGTCA
Hspb1 for CRISPR	GCGTCGCGCTCTCGAATTC	GTCTTGACCGTCAGCTCGTC
Nrf-2 for CRISPR	CTTCTCTTATATAAGCCAGTGGC	GCCACACACAGTAACGCCAG
Hspb1 (Human)	CTGACGGTCAAGACCAAGGATG	GTGTATTTCCGCGTGAAGCACC
Nrf2 (Human)	CACATCCAGTCAGAAACCAGTGG	GGA ATGTCTGCGCCAAAAGCTG
IL-1β (Human)	ATGATGGCTTATTACAGTGGCAA	GTCGGAGATTCGTAGCTGGA
IL-6 (Human)	CCTTCCAAAGATGGCTGAAA	TGGCTTGTTCCTCACTACT
NFκBIα (Human)	CTCCGAGACTTTCGAGGAAATAC	GCCATTGTAGTTGGTAGCCTTCA
ICAM1 (Human)	AGCGGCTGACGTGTGCAGTAAT	TCTGAGACCTCTGGCTTCGTCA
TNFα (Human)	CTTCTGGCTCAAAAAGAGAA	GTCAGGGATCAAAGCTGTAG
MMP3 (Human)	CGGTTCCGCCTGTCTCAAG	CGCCAAAAGTGCCTGTCTT
CXCL8 (Human)	CACTGCGCCAACACAGAAAT	TTCTCAGCCCTCTTCAAAAACTT
GAPDH (Human)	GAAGGTGAAGGTCGGAGTCA	TTGAGGTCAATGAAGGGGTC
